# Small RNA profiles from Panax notoginseng roots differing in sizes reveal correlation between miR156 abundances and root biomass levels

**DOI:** 10.1038/s41598-017-09670-8

**Published:** 2017-08-25

**Authors:** Yun Zheng, Kun Chen, Zhenning Xu, Peiran Liao, Xiaotuo Zhang, Li Liu, Kangning Wei, Diqiu Liu, Yong-Fang Li, Ramanjulu Sunkar, Xiuming Cui

**Affiliations:** 1 Faculty of Life Science and Technology, Kunming University of Science and Technology, Kunming, Yunnan 650500 China; 2 Yunnan Key Laboratory of Primate Biomedical Research, Institute of Primate Translational Medicine, Kunming University of Science and Technology, Kunming, Yunnan 650500 China; 3 Key laboratory of Panax notoginseng resources sustainable development and utilization of state administration of traditional Chinese medicine, Kunming, Yunnan 650500 China; 4 College of Life Sciences, Henan Normal University, Xinxiang, Henan 453007 China; 5 Department of Biochemistry and Molecular Biology, Oklahoma State University, Stillwater, Oklahoma USA

## Abstract

Plant genomes encode several classes of small regulatory RNAs (sRNAs) that play critical roles in both development and stress responses. *Panax notoginseng* (Burk.) F.H. Chen (*P. notoginseng*) is an important traditional Chinese herbal medicinal plant species for its haemostatic effects. Therefore, the root yield of *P. notoginseng* is a major economically important trait since the roots of *P. notoginseng* are the parts used to produce medicine. To identify sRNAs that are critical for the root biomass of *P. notoginseng*, we performed a comprehensive study of miRNA transcriptomes from *P. notoginseng* roots of different biomasses. We identified 675 conserved miRNAs, of which 180 pre-miRNAs are also identified, and three TAS3 loci in *P. notoginseng*. By using degradome sequencing, we identified 79 conserved miRNA:target or tasiRNA:target interactions, of which eight were further confirmed with the RLM 5′-RACE experiments. More importantly, our results revealed that a member of miR156 family and one of its SPL target genes have inverse expression levels, which is tightly correlated with greater root biomass contents. These results not only contributes to overall understanding of post-transcriptional gene regulation in roots of *P. notoginseng* but also could serve as markers for breeding *P. notoginseng* with greater root yield.

## Introduction

MicroRNAs are important small non-coding RNAs that play essential regulatory roles in plant development and stress responses^1–3^. Transcribed by RNA polymerase II^4^, the primary transcripts of miRNAs often form typical hairpin structures that are cleaved twice by Dicer Like proteins (DCL1) in nucleus^5^. After being exported to cytoplasm, miRNAs are loaded into an RNA-induced silencing complex (RISC) that normally contains an Argonaute (AGO) protein, and guide the RISC to cause site-specific cleavage or to repress the translation of mRNA targets^6, 7^.

Root tissue is derived from root apical meristem (RAM) that comprises highly active root initials and inactive quiescent center. Different layer of root system such as epidermis, cortex, endodermis, pericycle and vascular bundles originates from root initials firstly from mitotic divisions followed by expansion and differentiation^8^. Because plant miRNAs largely regulate diverse families of transcription factors, miRNAs in primary roots could function in gene regulatory networks that control root cell division, cell elongation, cell differentiation into various layers of root system and maintenance of the root apical meristem activity including quiescent center. Furthermore, root development is intimately linked with the integration of auxin-mediated gene regulation. In this context, miRNAs such as miR393 that regulates auxin receptor(s), TIR1 and closely related auxin-related F-box proteins (AFBs), whereas miR160, miR167 and miR390-dependent TAS3 siRNAs regulate 8 members of auxin response factors in *Arabidopsis* ^6, 9, 10^, thus, miRNAs are critical players in auxin signaling. Indeed, it was shown that miR160, by targeting ARF17, determines the primary root length in *Arabidopsis* ^11^. Similarly, miR164 regulation of NAC1 transcription factor is required for normal root development^12^. Furthermore, miR165/166 that regulate PHABULOSA (PHB) and PHAVULOTA (PHV) transcription factors and miR165/166-mediated repression of PHB/PHV genes is critical for proper embryonic root development^13^ and differentiation of the xylem^14^. Additionally, by targeting three SQUAMOSA PROMOTER BINDING PROTEIN-LIKE (SPL) genes, miR156 plays a role in lateral root development in *Arabidopsis* ^15^. Thus, miRNAs play critical roles in growth and development of plant roots^16–18^. It is interesting to note that miR156 overexpression could enhance shoot biomass accumulation as shown recently in switchgrass, a bioenergy plant species^19^.

*Panax notoginseng* (Burk.) F.H. Chen (*P. notoginseng*) is an important traditional Chinese herbal medicine and mainly grown in Yunnan province of China. Gensinosides, the unique medical compounds, are exclusively isolated from roots of Panax species. *P. notoginseng* had been cultivated for more than 400 years^20^ and used as medicine in China for its hemostatic properties^21^. The root of *P. notoginseng* is also used to produce Chinese herbal medicines, such as Yunnan Baiyao for curing trauma and bleeding^20^. Because the yield of *P. notoginseng* roots is of great medical, economic and social importance, it is interesting to scrutinize whether some miRNA and/or siRNAs play a role in the root yield of *P. notoginseng*. Thus far, there is only one published report on miRNAs in *P. notoginseng* ^22^ which only examined mature miRNAs. Without pre-miRNAs, it is difficult to estimate the number of conserved miRNAs, since a pre-miRNA often generates several isomiRs. Furthermore, studying miRNA populations in *P. notoginseng* plants differing in their root biomass contents could lead to identification of miRNA markers for better root yield.

Thus, we performed a research to identify miRNAs and siRNAs in *P. notoginseng* plants with varying root biomasses. By sequencing small RNAs from 17 root samples with varying root biomasses, we identified 675 conserved miRNAs, 72 putative novel miRNAs, and 3 TAS3 loci in *P. notoginseng*. Statistical analysis show that two members of the miR156 family have significantly higher expression levels in *P. notoginseng* roots with larger biomass contents. By sequencing a degradome library of *P. notoginseng* root, we identified 79 conserved and many non-conserved targets for the miRNAs and tasiRNAs. The expression levels of one member of miR156 family and one of its targets were significantly correlated with the root biomasses in *P. notoginseng*. These results provide a comprehensive view of sRNA transcriptomes and sRNA-guided regulatory networks in the root of *P. notoginseng* and offer new insights for identifying markers and breeding more productive *P. notoginseng*.

## Results

### Samples and characteristics of *P. notoginseng* plants

**Figure 1:**
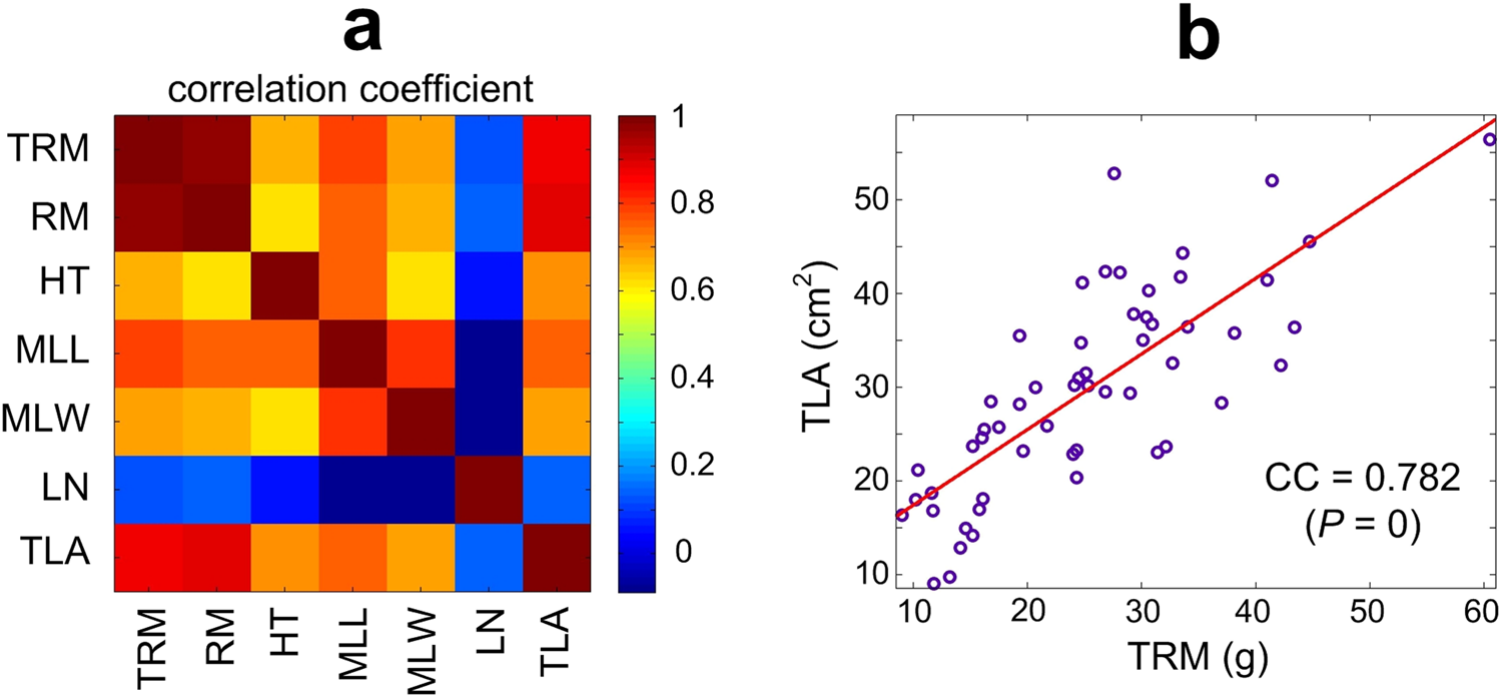
The correlation coefficients between characteristics of *P. notoginseng* plants. (**a**) The correlation coefficient matrix of Total Root Mass (TRM), Root Weight (RM), Height (HT), Middle Leaf Length (MLL), Middle Leaf Width (MLW), and Total Leaf Area (TLA). (**b**) The plot and correlation coefficient of TRW vs. TLA.

We collected 59 plants of *P. notoginseng* (see Supplementary Tables S1) grown in Wenshan County, Yunnan, China and recorded nine different attributes for these plants (as defined in Table S2), and calculated the correlation coefficients for 7 features. The results show that the Total Root Mass (TRM), the major parameter of yield, is significantly positively correlated with other five parameters, i.e., Root Mass (RM), Height (HT), Middle Leaf Length (MLL), Middle Leaf Width (MLW), and Total Leaf Area (TLA) (see Fig. 1a). For example, TRM and TLA have a significant correlation coefficient of 0.782 (*P* = 0) (Fig. 1b). These results indicate that plants with larger TLA could serve as a marker for higher root yields. Because TLA are not easily available, the MLL (with the second largest correlation coefficient) alternatively is a good indicative feature that could be used in practice.

### Small RNA profiles of *P. notoginseng*

Based on the integrity and quantity of the extracted RNAs, we selected 17 total RNA samples that belong to different groups of plants defined by the TRMs (see Materials and methods). The small RNAs of these 17 selected samples were sequenced using Illumina HiSeq. 2000 sequencer. Upon sequencing of these 17 small RNA libraries, each library yielded approximately 11 million qualified reads, thus in total, we obtained over 190 million qualified reads represented by more than 38 million unique reads. These reads were aligned to transcript database of *P. notoginseng*, non-coding RNAs, pre-miRNAs in miRBase (v21), and repeats (Table S3). The reads and unique sequences of 21 nucleotides (nt) and 24 nt are over-represented than those of other lengths (Figure S2a). But the reads/unique sequences mapping to pre-miRNAs only have a peak at 21 nt (Figure S2b).

### Conserved miRNAs in *P. notoginseng*

**Figure 2:**
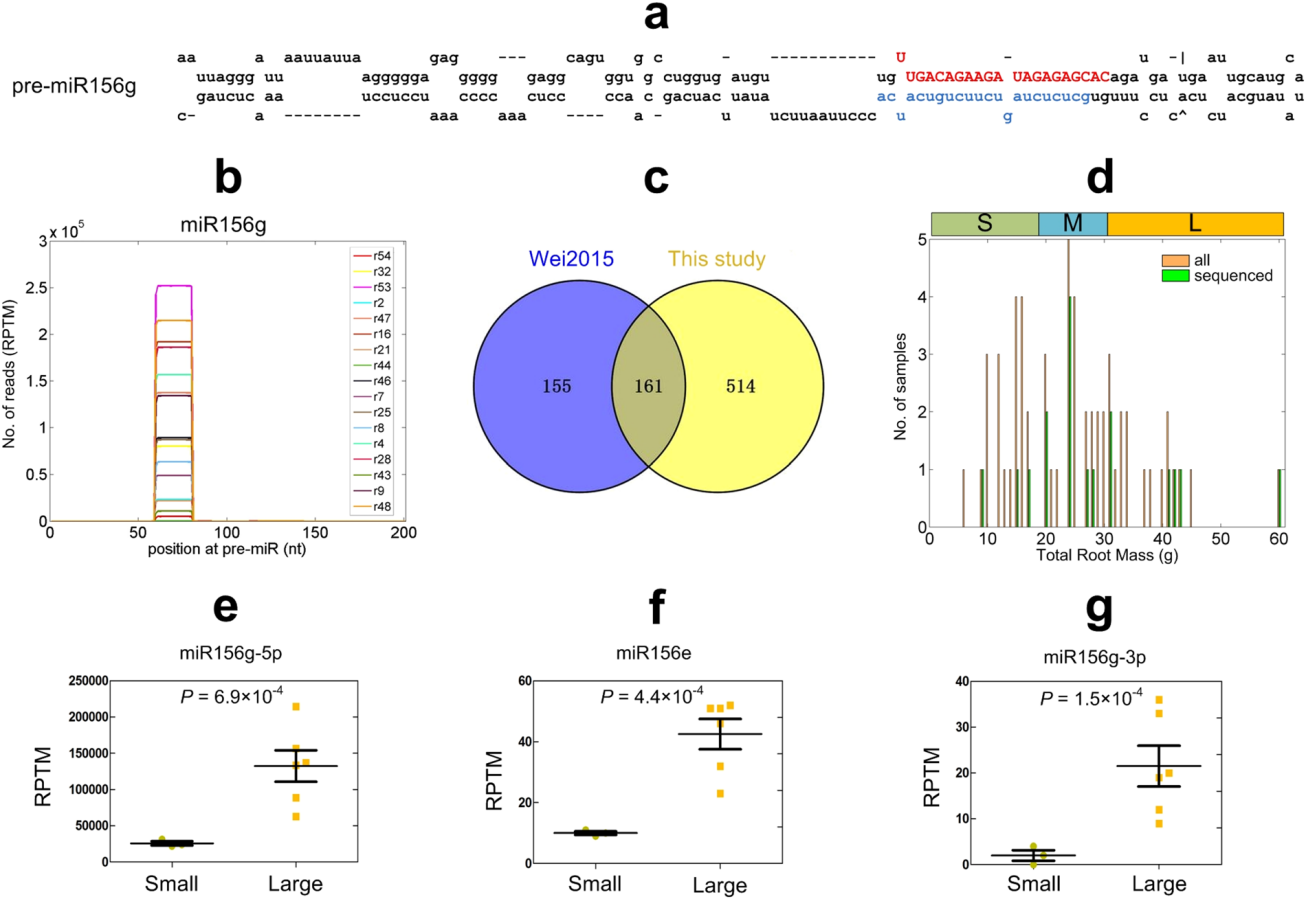
Conserved miRNAs of *P. notoginseng*. (**a**) The secondary structure of one of the identified pre-miRNAs, pre-miR156g. The red and blue parts are the mature miRNAs on the 5′ and 3′ arm of the pre-miRNA. (**b**) The distributions of small RNA reads on one of the identified pre-miRNAs, pre-miR156g in Part a. (**c**) The number conserved miRNAs reported in an existing study^22^ (in the circle of Wei2015) and this study. (**d**) The histogram of the numbers of samples with different Total Root Weights. S, M and L represent the Small, Medium, and Large groups, respectively. (**e**) to (**g**) The comparisons of the normalized abundances of miR156g2-5p, miR156e and miR156g-3p, respectively, in the Small and Large groups.

We were able to identify 180 pre-miRNAs of *P. notoginseng* after aligning the conserved miRNAs in other species to a transcript database of *P. notoginseng* (Table S4). In addition, we identified another 340 mature miRNAs by aligning the sRNA profiles to the mature miRNAs in the miRBase (v21) (Table S4). When compared to two model species, *Arabidopsis thaliana* and rice, we found 165 members that belong to 22 miRNA families that are highly conserved (Table 1). Fifty six precursors of these 165 miRNAs were identified (Table 1). The secondary structure and the distribution of reads of one of the newly identified pre-miRNAs are shown in Fig. 2a and b, respectively.

**Table 1:** Number of conserved miRNA genes in *P. notoginseng*.

miRNA family	*Arabidopsis*	Rice	*P. notoginseng*
miR156/529/535	10	12	30(5)
miR159	3	6	12(2)
miR160	3	6	5(6)
miR162	2	2	3(1)
miR164	3	6	7(3)
miR165/166	9	13	12(1)
miR167	4	10	6(4)
miR168	2	2	3(2)
miR169	14	18	14(4)
miR170/171	4	9	13(6)
miR172	5	4	7(6)
miR319	3	2	6(3)
miR390	2	1	6(0)
miR393	2	2	5(0)
miR394	2	1	1(0)
miR395	6	25	4(0)
miR396	2	8	11(5)
miR397	2	2	6(2)
miR398	3	2	3(1)
miR399	6	11	6(1)
miR403	1	0	3(2)
miR408	1	1	2(2)
sum	89	149	165(56)

In comparison with a previous report^22^ (see Fig. 2c), 161 conserved mature miRNAs overlapped between the published study^22^ and ours, but most importantly, 539 new miRNAs were identified in our study.

As shown in Fig. 2d, to identify miRNAs that may contribute to the biomasses of roots, the plant samples were classified into three groups, Small, Medium and Large based on the TRMs (details given in Materials and Methods). The miRNAs with at least 10 reads per ten million (RPTM) sequencing tags in either the Small or Large group were compared (Table S5). Interestingly, we found that two miR156 family members (MIR156g-5p and miR156e in Fig. 2e and f, respectively) show significantly higher expression levels in the Large group than in the Small group (multiple test corrected $P=6.9\times {10}^{-4}$ and $P=4.4\times {10}^{-4}$, respectively). MIR156g-3p is also more abundant in the Large group than in the Small group (multiple test corrected $P=1.5\times {10}^{-4}$, Fig. 2g).

### Novel miRNAs in *P. notoginseng*

**Figure 3:**
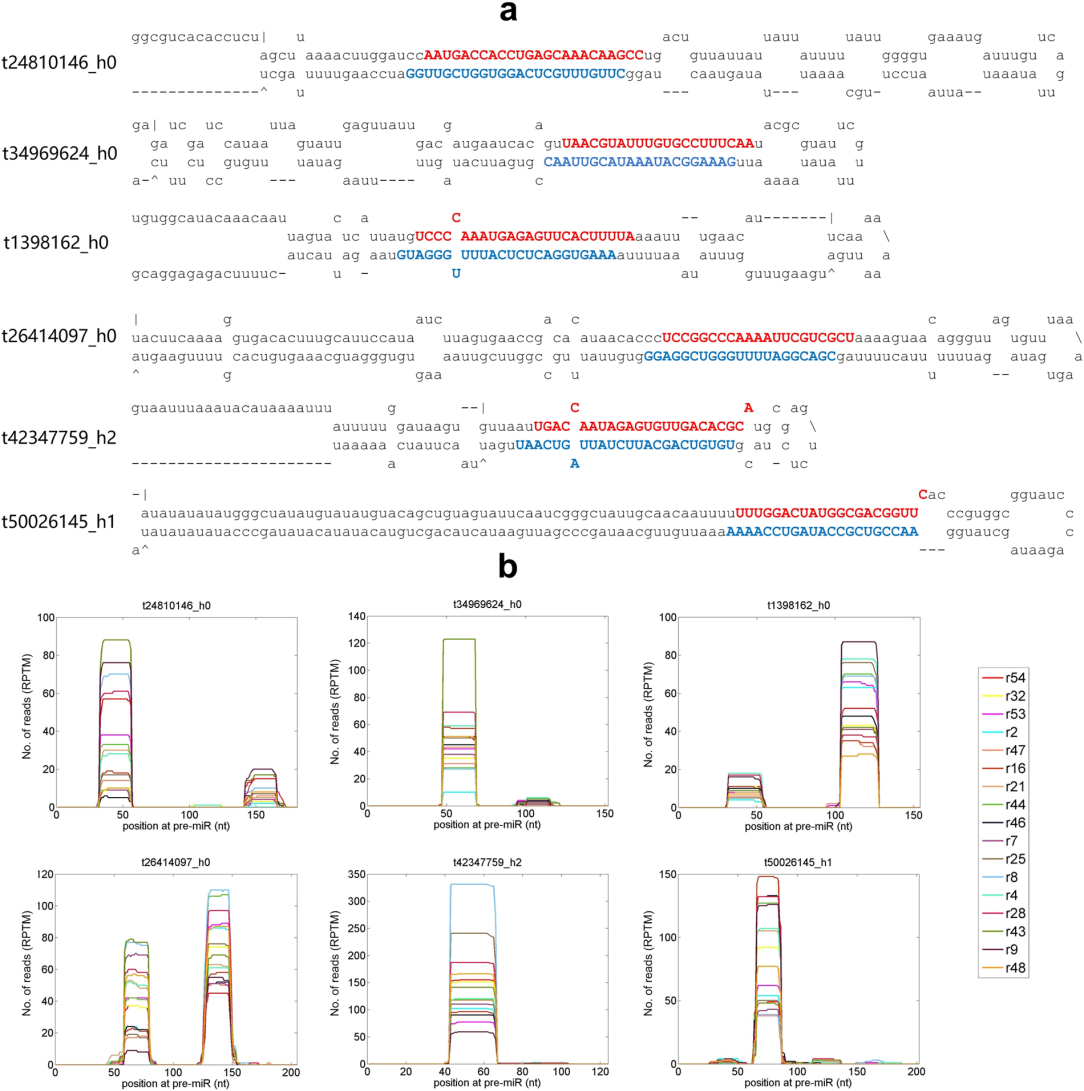
Some of the novel miRNAs of *P. notoginseng*. (**a**) The secondary structures of six novel miRNAs. (**b**) The distributions of small RNA reads on the novel pre-miRNAs in Part a.

We found 72 novel pre-miRNAs (Table S6), of which 6 examples were shown in Fig. 3. The precursors of these miRNAs form good hairpin structures (Fig. 3a) and the mature miRNAs on both the 5′ and 3′ arm of the hairpin structures are detected in the sequencing libraries. Figure 3b shows that most small RNAs generated from these pre-miRNAs are mature miRNAs on either 5′ or 3′ arms. Other novel miRNAs in Table S6 are similar to the 6 examples shown in Fig. 3. Only one novel miRNA has shown significantly lower expression level (multiple test corrected $P=2.9\times {10}^{-4}$) in the Large group (Table S7).

### The degradome of *P. notoginseng* root

After removing low quality reads from over 37 million raw sequencing reads, we obtained around 12.5 million qualified sequencing reads from the degradome of *P. notoginseng* root. The majority (87.7%) of these qualified reads could be mapped to the transcriptome of *P. notoginseng* root (Table S8), suggesting that the obtained degradome is of high quality.

### Conserved miRNA targets in *P. notoginseng*

**Figure 4:**
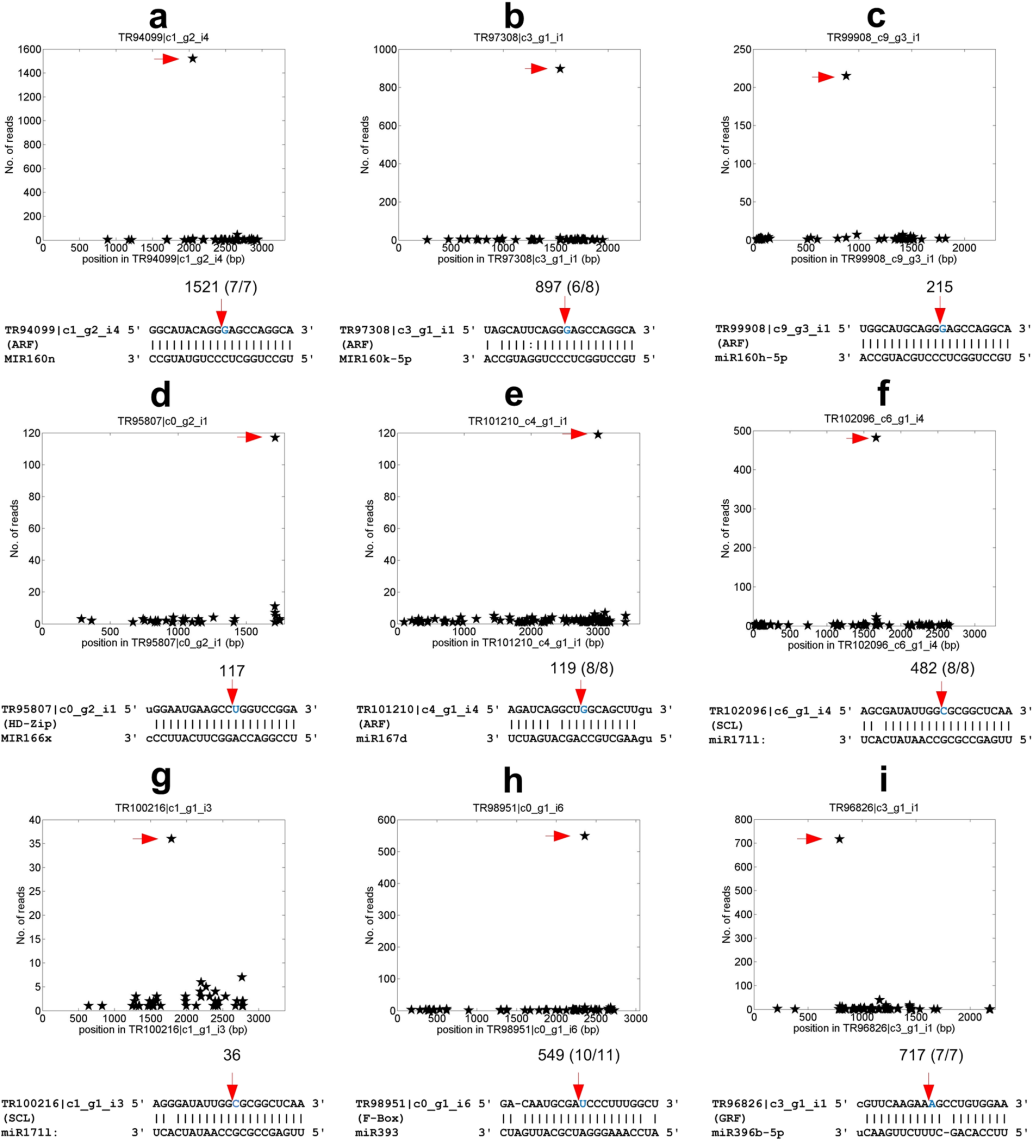
The t-plots and miRNA complementary sites of some conserved targets. The x-axis is the position on the transcript, and y-axis is the number of degradome reads detected from a position. The arrows in the upper parts correspond to the positions pointed by the arrows of the same colours in the lower parts. In Part (**a**) to (i), the numbers above the red arrows indicate the number of degradome reads from the position. In Part (**a**,**b**,**e**,**f**,**h**) and (**i**), the numbers in the parenthesis are the cleavage frequencies determined by the RLM 5′-RACE experiments. (**a**) MIR160n:TR94099 |c1_g2_i4 (an ARF gene). (**b**) MIR160k-5p:TR97308 |c3_g1_i1 (an ARF gene). (**c**) miR160h-5p:TR99908 |c9_g3_i1 (an ARF gene). (**d**) MIR166x:TR95807 |c0_g2_i1 (an HD-Zip gene). (**e**) miR167d:TR101210 |c4_g1_i4 (an ARF gene). (**f**) miR171l:TR102096 |c6_g1_i4 (an SCL gene). (**g**) miR171l:TR100216 |c1_g1_i3 (an SCL gene). (**h**) miR393:TR98951 |c0_g1_i6 (an F-Box gene). (**i**) miR396b-5p:TR96826 |c3_g1_i1 (a GRF gene).

We used the SeqTar algorithm^23^ for processing degradome reads and subsequently to identify targets that have been cleaved as a result of miRNA or TAS3-siRNA activity. This analysis revealed 79 conserved targets for 21 conserved miRNA families and TAS3 generated tasiARFs (see Table 2, Table S9 and Fig. 4). Three conserved miRNA:target relations, i.e., miR395:sulfate transporter, miR398:CCS1, and miR408:Plantacyanin were not found in *P. notoginseng*, probably due to non availability of the transcript information. Another reason could be the low expression levels of miR395, miR398 and miR408 in normal conditions since these three miRNA families are induced in stress conditions^3^. The miR156 family has five SPL targets in *P. notoginseng* (Table 2).

**Table 2:** Number of conserved miRNA targets in *P. notoginseng*.

miR family	Target gene family	*Arabidopsis*	Rice	*P. notoginseng*
miR156/529/535	SPL transcription factors	11	10	5
miR159/319	MYB transcription factors	7	2	5
miR159/319	TCP	5	4	1
miR160	ARF transcription factors	3	4	5
miR162	DCL	1	1	1*
miR164	NAC transcription factors	7	6	5
miR165/166	HD-Zip transcription factors	6	4	8
miR167	ARF transcription factors	2	4	3
miR168	Argonaute	1	6	1
miR169	HAP2 transcription factors	7	8	4
miR170/171	SCL transcription factors	4	5	5
miR172	AP2 transcription factors	6	5	9
miR390/391	TAS3	3	3	3
miR393	F-Box	5	2	3
miR394	F-Box	1	1	1
miR395	SO_2_ Transp.	1	3	0
miR395	APS	3	1	1
miR396	GRF	7	12	6
miR397	Laccase	3	16	2
miR398	CSD	2	2	1*
miR398	CCS1	1	1	0
miR399	PO_4_ Transp.	1	4	3
miR399	E2-UBC	1	1	1*
miR403	Argonaute 2	2	0	1*
miR408	Laccase	3	2	1
miR408	Plantacyanin	3	7	0
TAS3-siR	ARF	3	5	4
sum		99	119	79

*means the numbers include targets with 4 to 5 mismatches.

Twenty two conserved miRNA:target pairs were verified with the degradome reads (with at least 1 valid degradome read, as shown in Table S9). For examples, miR160 family targets three ARF family members (Fig. 4a–c, respectively); miR166x targets one HD-Zip family member (Fig. 4d); miR167d targets an ARF family member (Fig. 4e); miR171 family targets two SCL family members (Fig. 4f and g, respectively); miR393 targets an F-box family member (Fig. 4h) and miR396 targets a GRF family member (Fig. 4i).

We chose ten of the 79 conserved targets for further validation using RLM 5′-RACE experiments (Table S10). Eight of these 10 selected targets were successfully validated, of which six were shown in Fig. 4a,b,e,f,h and i, respectively. One SPL gene, TR103070 |c5_g1_i1, targeted by miR156f and one GRF gene, TR53089 |c0_g1_i1, targeted by MIR396n, were also confirmed in the RLM 5′-RACE experiments (with 10/10 and 5/5 cleavage frequencies, respectively) (see Figure S3).

### Other targets of conserved miRNAs

**Figure 5:**
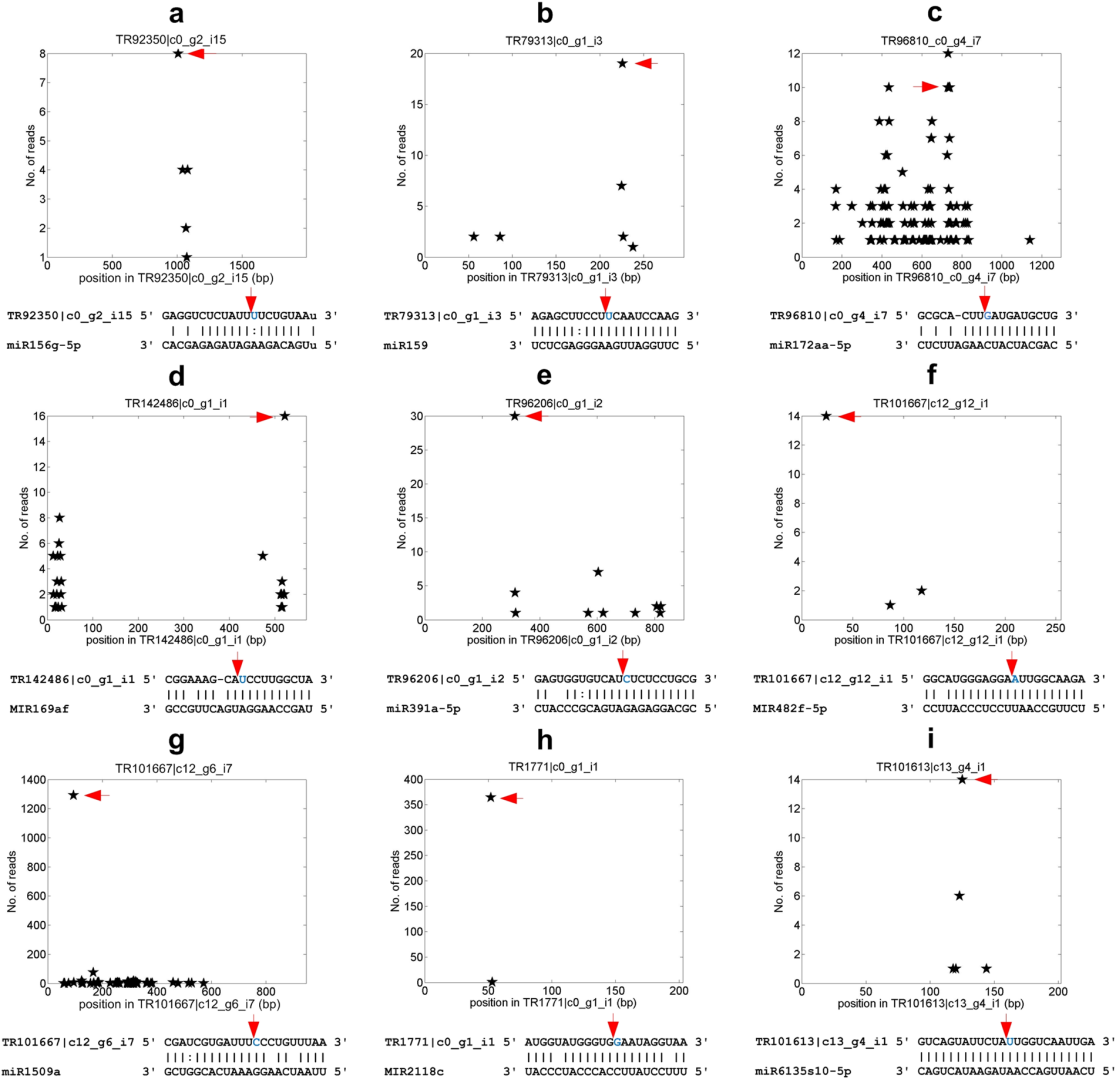
The t-plots and miRNA complementary sites of some non-conserved targets of conserved miRNAs. (**a**) miR156g-5p:TR92350 |c0_g2_i15 (a putative ribokinase gene). (**b**) miR159:TR79313 |c0_g1_i3 (an unknown gene). (**c**) miR172aa-5p:TR96810 |c0_g4_i7 (a putative ADP-ribosylation factor 3 gene). (**d**) MIR169af:TR142486 |c0_g1_i1 (a putative TIFY 6B gene). (**e**) miR391a-5p: TR96206 |c0_g1_i2 (a putative calcium-transporting ATPase 10, plasma membrane-type, gene). (**f**) MIR482f-5p: TR101667 |c12_g12_i1 (an unknown gene). (**g**) miR1509a:TR101667 |c12_g6_i7 (an unknown gene). (**h**) MIR2118c: TR1771 |c0_g1_i1 (a TMV resistance protein N-like gene) (**i**) miR6135s10-5p: TR101613 |c13_g4_i1 (an unknown gene).

In addition to the conserved targets, conserved miRNAs are known to targeting additional genes in diverse plant species. Thus, we also examined potential novel targets for conserved miRNAs. This scrutiny has identified over thirty seven thousand targets for six hundred conserved miRNAs (Table S11). More than one hundred of these targets were reliably confirmed by the degradome sequencing profile, with at least 10 valid reads (Table S11). Nine of these targets were shown in Fig. 5, i.e., miR156g-5p targets TR92350 |c0_g2_i15, a putative ribokinase gene (Fig. 5a); miR159 targets TR79313 |c0_g1_i3, an unknown gene (Fig. 5b); miR172aa-5p targets TR96810 |c0_g4_i7, a putative ADP-ribosylation factor 3 gene (Fig. 5c); MIR169af targets TR142486 |c0_g1_i1, a TIFY 6B gene (Fig. 5d); miR391a-5p targets TR96206 |c0_g1_i2, a putative calcium-transporting ATPase 10, plasma membrane-type, gene (Fig. 5e); MIR482f-5p targets TR101667 |c12_g12_i1, an unknown gene (Fig. 5f); miR1509a targets TR101667 |c12_g6_i7, an unknown gene (Fig. 5g); MIR2118c targets TR1771 |c0_g1_i1, a putative TMV resistance protein N-like gene (Fig. 5h); and miR6135s10-5p targets TR101613 |c13_g4_i1, an unknown gene (Fig. 5i).

miR1507, miR1509 and miR2118 are 22 nt miRNAs that initiate the productions of phased siRNAs by targeting NB-LRR disease resistance genes, transcription factor, and NB-LRR genes, respectively, in *Medicago truncatula* ^24^. In addition to legumes, these families were conserved in other plant species^24^. All these three families were detected in our sequencing libraries (Table S4), indicating these three families were conserved in *P. notoginseng* as well. In addition to TR101667 |c12_g6_i7 shown in Fig. 5g, eight other unknown genes were shown to be targeted by miR1509 in *P. notoginseng* with strong cleavage effects (Table S11). The miR2118c target in Fig. 5h is putative TMV resistance protein, suggesting that it is a potential NB-LRR disease resistance gene.

TR101613 |c13_g4_i1 in Fig. 5i is a putative *cis*-target of miR6135s10-5p because this miRNA is encoded by the same gene. Previously, it has been noticed that several conserved miRNAs cleave their own primary transcripts^10^. Our results suggest that self-repressions exist even in non-conserved miRNAs.

These results suggest that conserved miRNAs may have other functions than targeting the conserved targets shown in Table 2.

### Validating expression patterns of miR156g-5p and its targets with qRT-PCR

**Figure 6:**
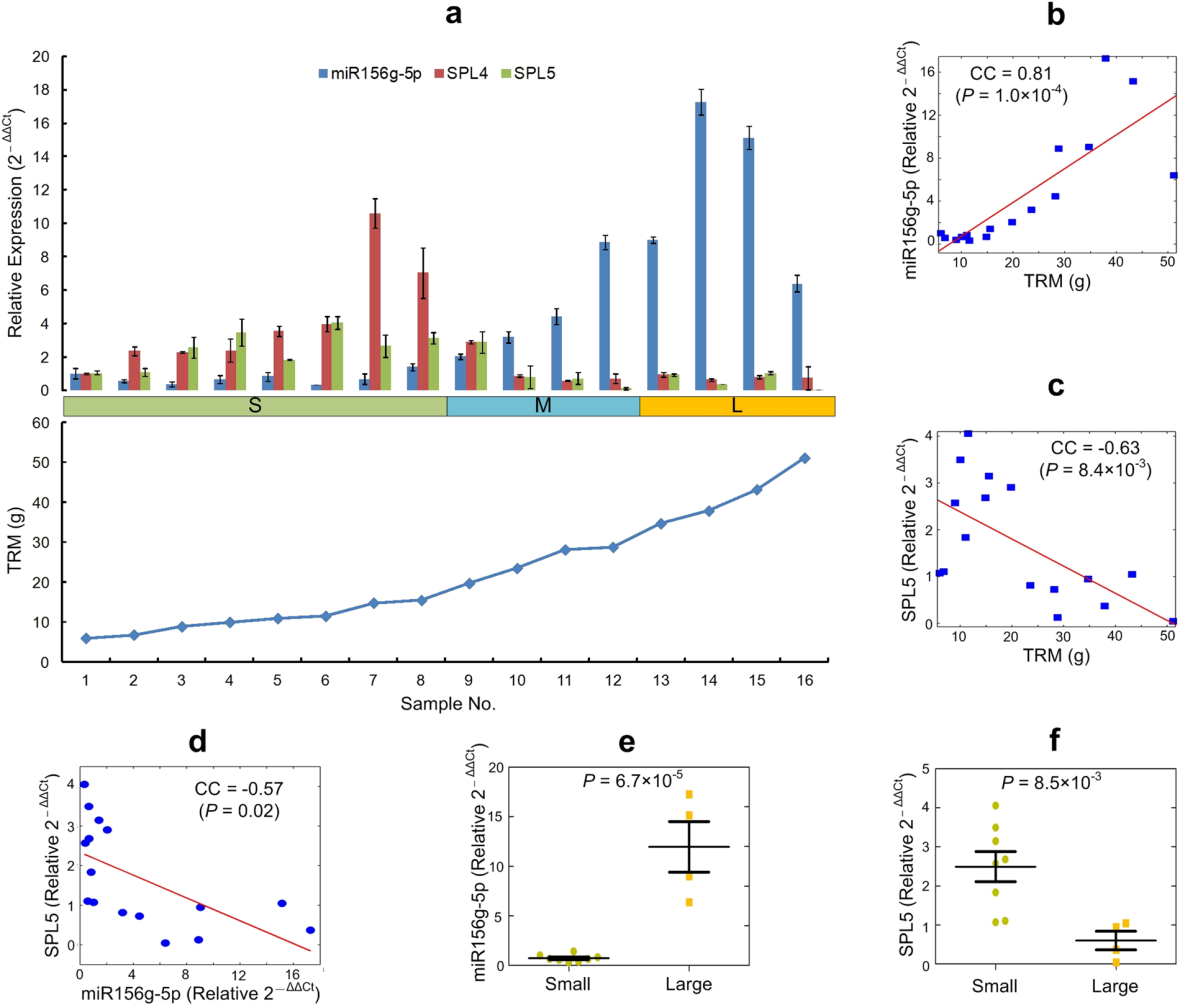
Validating expression patterns of miR156g-5p and its two SPL target genes with qRT-PCR. (**a**) The relative expressoin levels of miR156g-5p and two SPL target genes, and the TRMs of 16 samples. SPL4 and SPL5 are TR75240 |c1_g1_i1 and TR94274 |c1_g1_i1, respectively. The bars and error bars represent the mean values ± standard deviations. S, M and L represent the Small, Medium, and Large groups, respectively. (**b**) The relation between the expression level of miR156g-5p and TRMs of the samples. (**c**) The relation between the expression level of SPL5 and TRMs of the samples. (**d**) The relation between the expression levels of miR156g-5p and SPL5. (**e**) The comparison of the expression levels of miR156g-5p in the Small and Large groups. (**f**) The comparison of the expression levels of SPL5 in the Small and Large groups.

To further validate the roles of miR156g-5p and its targets in accumulation of root biomass, we collected 16 *P. notoginseng* roots with different TRMs and examined the expression levels of miR156g-5p and two SPL genes that are targeted by miR156g-5p. These 16 root samples were also classified into three groups using the same criteria as the samples for sRNA sequencing profiles. The obtained results were shown in Fig. 6. miR156g-5p has shown gradually increasing expression levels when the TRM of the samples increases (Fig. 6a), while the two SPL genes have shown gradually decreasing levels. The correlation coefficient between the expression levels of miR156g-5p and SPL5 and the TRMs of the samples are significant positive and negative ($P=1.0\times {10}^{-4}$ and $8.4\times {10}^{-3}$, in Fig. 6b and c, respectively), respectively. SPL4 also has a negative correlation coefficient, −0.43, with the TRM of the sample, but insignificant ($P=0.096$). The expression levels of miR156g-5p and SPL5 is also significantly negatively correlated (*P* = 0.02, Fig. 6d), indicating that miR156g-5p represses SPL5. miR156g-5p and SPL5 has shown significantly higher and lower expression levels in roots of larger biomasses ($P=6.7\times {10}^{-5}$ and $8.5\times {10}^{-3}$, *t*-test, Fig. 6e and f, respectively), respectively. The other target, SPL4, has lower expression levels in roots with larger biomasses but slightly insignificant ($P=0.065$, *t*-test). These results suggest that miR156g-5p does contribute to the increases of the root biomasses by repressing SPL5, as well as SPL4 at a less significant level.

### Targets of non-conserved novel miRNAs

We predicted 162 targets for the novel non-conserved miRNAs (Table S12). Four of the predicted targets of novel miRNAs were shown in Figure S3, i.e., t7697358 targets TR52460 |c0_g4_i1, a putative 60S ribosomal protein gene (Figure S4a); t8973631 targets TR97626 |c1_g2_i7, an unknown gene (Figure S4b); t40632331 targets TR97419 |c0_g1_i2, an unknown gene (Figure S4c); and t33730249 targets TR101500 |c9_g5_i1, a 40S ribosomal protein Sa-2-like gene (Figure S4d). TR97626 |c1_g2_i7 is a putative anti-sense target of t8973631, which is encoded by anti-sense strand of TR97626 |c1_g2_i6.

### TAS3 and tasiRNAs in *P. notoginseng*

**Figure 7:**
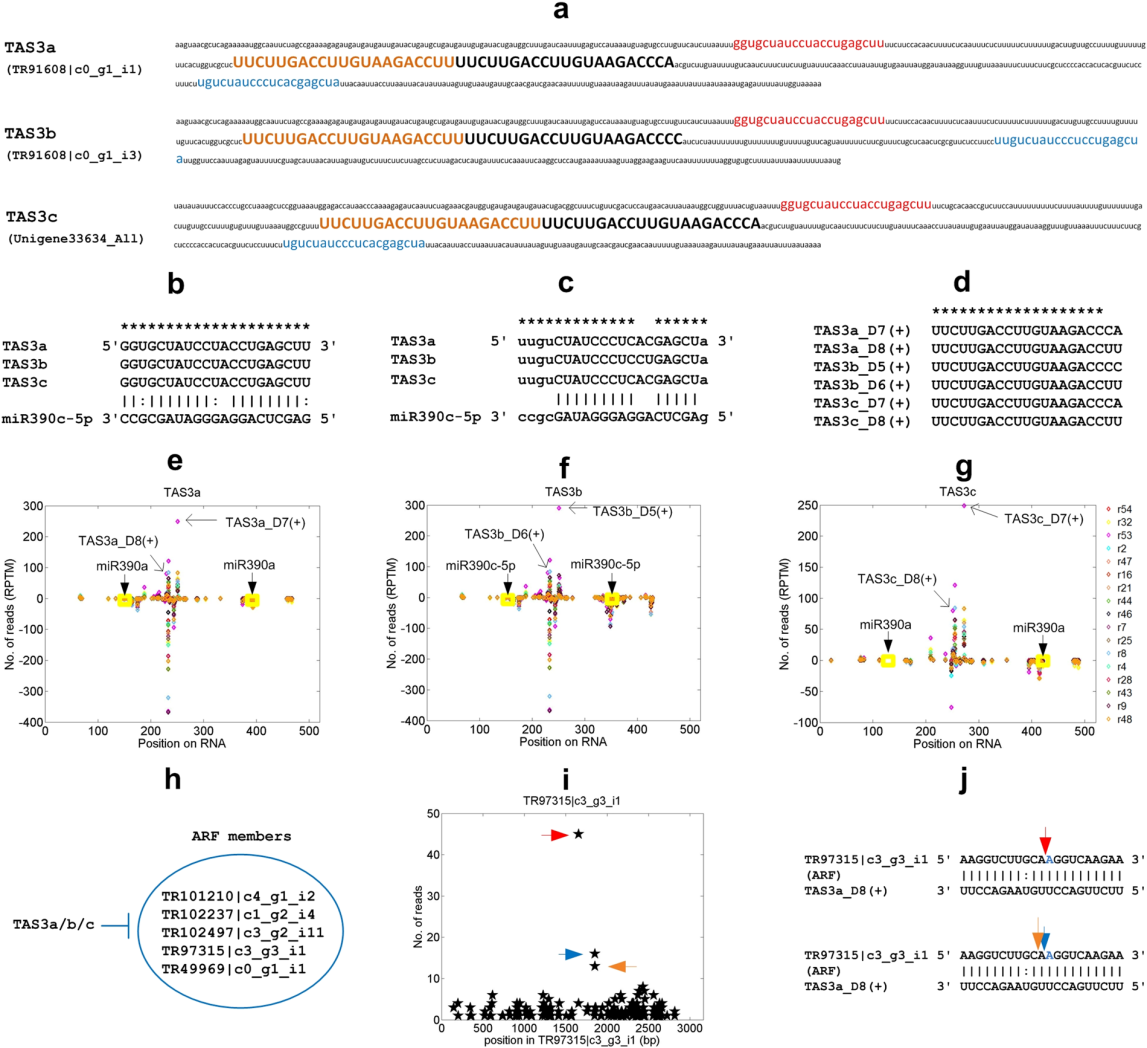
The three identified TAS3 loci and their encoded tasiRNAs in *P. notoginseng*. (**a**) The sequences of the three putative TAS3 loci in *P. notoginseng*. The red and blue regions are 5′ and 3′ miR390 complementary sites, respectively. The regions of upper case nucleotides are mature tasiRNAs that target ARF family members, or tasiARFs. (**b**) The 5′ miR390 binding sites on TAS3 transcripts. Only the commonly aligned nucleotides are aligned to miR390. (**c**) The 3′ miR390 binding sites on TAS3 transcripts. (**d**) The mature tasiRNAs that target ARF family members derived from TAS3a/b/c loci. (**e**) to (**g**) The distribution of small RNA reads in the three TAS3 loci. (**h**) The ARF genes that are targeted by tasiARFs encoded by TAS3a/b/c. (**i**) The t-plots of one of the ARF genes, TR97315 |c3_g3_i1, that is targeted by tasiARFs. The arrows in the this part correspond to the positions pointed by the arrows of the same colours in the Part j. (**j**) The two TAS3a_D8(+) complementary sites on TR97315 |c3_g3_i1.

We found three TAS3 loci in *P. notoginseng* (Fig. 7). These three TAS3 loci have two typical miR390 complementary sites around the conserved tasiRNAs (Fig. 7a–c). Each of these three TAS3 encodes two conserved tasiRNAs that target the ARF family genes, i.e., the tasiARFs (Fig. 7a and d). The second tasiARFs, from the 3′ miR390 site, encoded by these three TAS3 loci are the same, and the first tasiARFs are different only at the last nucleotide (Fig. 7d).

These three TAS3 loci generate many small RNAs (Fig. 7e–g). Among them, the conserved tasiARFs are highly expressed (Fig. 7e–g). The expression levels of tasiARFs in the Small and Large groups have no significant differences.

These tasiARFs generated by these three TAS3 loci target five ARF family genes (Fig. 7h and Table 2). One of the ARF family member, TR97315 |c3_g3_i1 in Fig. 7i, were verified by the degradome sequencing profile and cleaved by tasiARFs at two different sites (Fig. 7i and j).

The three TAS3 loci of *P. notoginseng* are closely related to TAS3 loci in several dicot species, such as *Nicotiana tabacum*, *Solanum lycopersicum* and *Olea europaea* (Figure S5a). The tasiARFs encoded in the three TAS3 loci of *P. notoginseng* is also close to the tasiARFs of dicot plant species (Figure S5b and c).

In addition to the three TAS3 targeted by miR390, we also found that MIR2118c targets two putative NB-LRR genes, TR1771 |c0_g1_i1 (Fig. 5h) and TR96289 |c1_g1_i1 (Table S11). As shown in Figure S6, these two transcripts could generate phased siRNAs (phasiRNAs) ($P=2.1\times {10}^{-4}$ and $3.3\times {10}^{-4}$, Hypergeometric test, respectively, see Materials and methods for details). The MIR2118c complementary sites on both transcripts initiate the generations of the 21 nt phasiRNAs, and the phase scores of the two transcripts are larger than 5 (Figure S6a and b) (see Materials and methods for details). TR96289 |c1_g1_i1 is also targeted by miR482 at a complementary site that is highly overlapped with the MIR2118c complementary site (Figure S6c).

## Discussion

Our comparative miRNA profiles in roots of varying sizes (small, medium and large) clearly revealed the roles of miRNAs, particularly the miR156 family, in root biomass accumulation in *P. notoginseng*. The abundances for miR156g-5p and miR156e varied significantly between the roots with different sizes. Roots with smaller size have lower abundances while the roots with larger size have extremely high abundances (Fig. 2). In general the sequencing-based profiles are trustworthy but there are instances where sequencing profiles differed from the qRT-PCR or even small RNA blot analysis. Therefore we independently validate these findings with qRT-PCR experiments. Our qRT-PCR analysis also indicated that the expression levels of miR156g-5p and SPL5 have significant positive and negative correlation coefficients ($P=1.0\times {10}^{-4}$ and $8.4\times {10}^{-3}$, in Fig. 6b and c, respectively), respectively, with the TRMs of the samples. During phase changes, i.e., from vegetative to reproductive growth, in leaves of *Arabidopsis*, miR156 expression decreases, and SPL expression increases^25^. In consistent with this, the expression level of miR156g-5p is negatively correlated with that of its target, SPL5, in *P. notoginseng* roots (Fig. 6d).

In shoots, genes specifically transcription factors and miRNAs form a network and coordinate the process of transition from vegetative to reproductive phase^26^. In this network, the suppression of miR156 is critically important, which allows surge in the abundances of SPL transcription factors whose activity is critical for turning-on several genes to initiate the reproductive phase^26^. Given the critical role of miR156 in suppressing SPL genes, miR156 has been exploited to enhance biomass accumulation of switchgrass^19^, a critical target trait in bioenergy plants. Similar results were also obtained in *Arabidopsis*, rice and maize^17^. Although, the exact mechanism involved in shoot biomass enhancement in miR156 over-expressing plants is unknown other than the suppression of SPL genes, which in turn could be suppressing the genes required for transiting into flowering phase. By contrast to the shoot phenomenon, the contributing factors for root biomass is largely unknown. More recent studies have suggested that miR156 regulated SPL genes repress adventitious root development in *Arabidopsis* ^15, 27^.

Other pertinent studies that support our reported findings here include tuber development in potato^28, 29^. Although potato tubers are not roots per se but a role for miR156 in tuber development is unequivocally established^28, 29^. More over a recent study that reported miRNA profiles from roots of *P. notoginseng* also showed that miR156 levels were the most highly expressed relative to other miRNA families^22^. They also reported that a few members of miR156 were most abundant in roots compared to shoots and leaves of *P. notoginseng* ^22^. Taken together, all these prior direct and indirect evidences as well as our findings that miR156 levels greatly differed between roots of different sizes imply that miR156 could also contribute to the root biomass accumulation, which can be exploited for improving root biomass contents using miR156 overexpression strategy. Because miR156 represses SPL genes, the strategies such RNAi or CRISPR-mediated silencing of SPL genes could offer as alternative approaches to enhance root biomass. These and other possibilities need worth exploring in future not only in this plant species but also in other root crops.

NB-LRR disease resistance genes that are targeted by the miR482/miR2118 superfamily could generate phasiRNAs in fabaceae and several other families^24, 30, 31^. We found that there are miR2118 genes in *P. notoginseng* and NB-LRR genes targeted miR2118 and miR482 could generated phasiRNAs, indicating that the phasiRNAs generation system initiated by miR482/miR2118 induced cleavage on NB-LRR transcripts is also conserved in Araliaceae, and is more widely conserved than previously reported.

miRNAs, such as miR160 and miR393, may participate in the disease resistance of plants^18^. Our results suggest that miR2118 might play a role in disease resistance in *P. notoginseng* through its regulation of the NB-LRR genes, in addition to miR160 and miR393.

One existing study compared differently expressed miRNAs in *P. notoginseng* roots at different ages^22^. In comparison, we explored miRNAs that have different expressed levels in roots of different biomasses but at the same age. When compared with this report^22^, our work have significantly improve the understanding of miRNAs in *P. notoginseng* in several aspects. First, we found additional conserved miRNAs. As shown in Fig. 2c, we found 514 conserved miRNAs that have not been reported previously. Second, 180 precursor sequences of conserved miRNAs were also found in this study while no precursor sequences were reported in previous studies. Third, we employed degradome sequencing to confirm some targets of conserved miRNAs and novel miRNAs. Fourth and the most importantly, we verified that miR156g-5p and one of its targets, SPL5, had significantly higher and lower expression levels in *P. notoginseng* roots with larger biomasses (Figs 2e, 6e and f), respectively. The expression level of miR-156g-5p is significantly negatively correlated with that of SPL5 ($P=0.02$, Fig. 6d). These results provide evidence that miR-156g-5p does contribute to the increases of the root biomasses in *P. notoginseng* by repressing the SPL5 gene.

## Conclusion

We identified 675 conserved miRNAs and seventy two novel miRNAs in *P. notoginseng* by sequencing the small RNAs of 17 *P. notoginseng* roots. Three TAS3 loci were identified in *P. notoginseng*. Seventy nine conserved targets of conserved miRNAs and TAS3 derived tasiRNAs were identified by using the degradome sequencing and the SeqTar algorithm. Eight of these 79 targets were further validated using the RLM 5′-RACE experiments. These results significantly improved our understanding of miRNA-guided gene regulatory networks in *P. notoginseng*. Ever more importantly, our results demonstrated that two miR156 members have significantly higher expression levels in *P. notoginseng* roots of larger biomasses, indicating their potential usages as biomarkers for selecting *P. notoginseng* strains with larger root biomasses. One SPL target of miR156g-5p, SPL5, has significantly lower expression levels in roots of larger biomasses. We also show that the expression level of miR156g-5p is positively correlated with the TRMs of *P. notoginseng*, while the expression level of SPL5 is negatively correlated with the TRMs, suggesting that miR156g-5p contributes to the increased TRMs of *P. notoginseng* by repressing SPL5.

## Materials and methods

### Samples and small RNA sequencing

Fifty nine *P. notoginseng* plants grown in Wenshan County, Yunnan, China were selected in the study (Table S1). Nine characteristics (defined in Table S2 and Figure S1), i.e., Total Root Mass (TRM), Root Mass (RM), Height (HT), Middle Leaf Length (MLL), Middle Leaf Width (MLW), Number of Complex Leaves, Total Leaf Area (TLA), Leaf Number (LN), and Leaf Pattern of the plants were measured and recorded.

The roots collected from 59 different *P. notoginseng* plants were frozen in liquid nitrogen immediately. The samples were stored at −80 °C until RNAs were extracted. Total RNAs were extracted from root tissues using the Trizol reagent (Invitrogen, Thermo Fisher Scientific Inc., USA) according to the manufacturer′s protocol. The integrities of the RNAs were checked using an ultraviolet spectrophotometer (Hoefer, MA, USA), based on the ratio of the optical density at 260 nm to that at 280 nm (OD260/280) and were also assessed by electrophoresis in a denaturing formaldehyde agarose gel, based on visual comparison with the 18S and 28S ribosomal RNAs. The RNA samples with OD260/280 between 1.8 and 2.0 were checked for the total quantities. Only samples with at least 20 *μ*g were chosen for preparation of sRNA sequencing libraries. The small RNAs of the 17 qualified samples were isolated from total RNAs and were sequenced using Illumina HiSeq. 2000 sequencer. The 17 obtained small RNA sequencing profiles had been deposited into the NCBI SRA database under the series accession number SRP082250.

### Analysis of the small RNA sequencing profiles

The obtained small RNA libraries were analyzed as described previously^32–34^. In brief, a unique count was created for each sequence after removing redundant reads. Then unique sequences mapped to known non-coding RNAs (rRNAs, tRNAs, snRNAs, snoRNAs) and repeats were removed from unique RNAs by aligning to databases Rfam (r11)^35^, NONCODE (v3.0)^36^, GtRNAdb^37^, Plant Repeat Databases^38^ and Repbase (r20)^39^ using SOAP2^40^. Remaining reads were mapped to the miRBase database (r21)^41^ to calculate the frequencies of conserved miRNAs. After this step, the remaining unique reads were aligned to a self-assembled transcript database of *P. notoginseng* (Zheng and Cui, unpublished). This Transcriptome Shotgun Assembly project has been deposited at DDBJ/EMBL/GenBank under the accession GFRX00000000. The version described in this paper is the first version, GFRX01000000. The flanking regions (150 nt down stream and 150 nt upstream) of the matched loci were used to predict fold-back structures using RNAfold^42^. Then, the structures with at least 18 paired nucleotides and a folding energy of smaller than −40 Kcal/mol were kept as putative precursors for further analysis. Next, the small RNA reads were mapped to the obtained putative precursors. Finally, novel miRNA identification and annotation were strictly based on appearance of miRNA* and predictable fold back structures for the miRNA precursor sequences, as suggested by Meyers *et al*.^43^.

### Degradome sequencing profile and processing

*P. notoginseng* plants used in the degradome sequencing profile and the qRT-PCR experiments were grown in a shading greenhouse at Kunming University of Science and Technology (24°51′0′′ N, 102°52′2′′E, altitude 1835 m), Kunming, Yunnan, China. During the experiments, the daily average temperature was 25 °C, the daily maximum difference in temperature was 10 °C and humidity was 60–80%.

The root of a *P. notoginseng* plant was frozen in liquid nitrogen immediately after harvesting. The total RNA from the root of a *P. notoginseng* plant was extracted using the Trizol reagent (Invitrogen, Thermo Fisher Scientific Inc., USA) according to the manufacturer′s protocol. The integrity of the RNA was checked with an ultraviolet spectrophotometry (Hoefer, MA, USA) and 2100 BioAnalyzer (Agilent Technologies, Santa Clara, CA, USA). The degradome of polyadenylated transcripts was sequenced using Illumina HiSeq. 4000 sequencer. The obtained sequencing profile had been deposited into the NCBI SRA database under the series accession number SRP087606. The obtained degradome sequencing profile was filtered to remove low quality reads that have low scored nucleotides (<20). Then, the 3′ adapters in the remaining reads were removed.

### Identification of conserved miRNAs

As described previously^33^, mature miRNA sequences from all plant species downloaded from the miRBase (v21)^41^ were combined to obtain unique miRNA sequences. Next, these unique miRNA sequences were used as queries against the self-assembled *P. notoginseng* transcript database using BLASTN to predict known miRNA homologs in *P. notoginseng* that were not represented in small RNA libraries. Hits with no more than two mismatches were identified and the flanking regions (150 nt down stream and 150 nt upstream) to the mapped mature miRNAs were isolated and used to predict fold-back structures using the RNAfold^42^. The predicted fold-back structures were examined for the presence of miRNA on the same arm of the hairpin as the known family members from other plants. These precursor sequences were further evaluated by MIRcheck^44^ and selected candidates that have ≤6 mismatches, ≤2 bulged or asymmetrically unpaired nucleotides, and ≤3 continuous mismatches within the mature miRNA.

We compared the identified conserved miRNAs with those reported previously^22^. If the sequences of a mature miRNA was the same as reported earlier^22^, the conserved miRNA was named the same as reported previously^22^. The remaining conserved miRNAs were named by using upper case MIR followed by the family name, and alphabetical letters in lower case if these have not been reported earlier^22^.

The numbers of conserved miRNA family members were counted if mature miRNAs were detected in the sequencing libraries. For mature miRNAs without pre-miRNAs, we count the numbers of conserved mature miRNAs that target the conserved gene families. If two mature miRNAs only have one or two different nucleotides at 5′ or 3′ end, they were regarded as the same member of a same miRNA family if the pre-miRNAs of these miRNAs were not available.

### Identifying the deregulated miRNAs in roots of different biomasses

All 59 selected samples were assigned to three groups based on their Total Root Masses (TRMs). The samples in the Medium group have TRM values within mean value (*m*) minus and plus 0.5 × standard deviation (*s*) of the 59 TRM values. The samples in the Small and Large group have TRM values smaller than *m* − 0.5 × *s* and larger than *m* + 0.5 × *s*, respectively. The Groups of the samples were listed in Table S1. The raw abundance of miRNAs were normalized to Reads Per Ten Million (RPTM) sequencing reads. The miRNAs with a mean abundance of at least 10 RPTM in either the Small or the Large group were selected. Then, the normalized abundances of selected miRNAs were compared with edgeR^45^. miRNAs with multiple-test corrected *P*-values of smaller than 0.05 were regarded as significantly deregulated miRNAs in the Small and Large group.

### Identification of TAS3 loci in *P. notoginseng*

As reported previously^46^, TAS3 derived tasiARFs, i.e., siRNAs that target the ARF family genes^47, 48^, from *Arabidopsis* and rice were aligned to the self-assembled transcript database of *P. notoginseng*, then we examined the typical miR390 complementary sites around the matched loci.

For miR2118 targets, we checked whether the downstream regions of the miR2118 complementary sites generated phased 21 nt siRNAs. A self-written program was used to scan the regions till the end of the transcripts using a window of 210 nt (ten 21 nt phases). A two-nucleotide positive offset was used to calculate the positions of siRNAs on the anti-sense strand because the existence of two-nucleotide over-hang at the 3′-end of siRNA duplex^24, 31, 34, 49–51^. Then a *P*-value was calculated for each of the windows using a modified version of methods in ref. 51,

1 $$P(X=k)=\sum _{X=k}^{m}\frac{(\begin{array}{c}20m\\ n-k\end{array})(\begin{array}{c}m\\ k\end{array})}{(\begin{array}{c}21m\\ n\end{array})},$$

where *n* was the number of unique 21 nt sRNAs mapped within a window, *k* was the number of phased unique 21 nt sRNAs within the window, and *m* was the number of phases. *m* was set to 10 in this study.

And a phase score was calculated for each position of the regions using the method in ref. 52. For a window started at a position with more than three phased unique sRNAs, i.e., when *k* ≥ 3,

2 $$PhaseScore={\rm{l}}{\rm{n}}{(1+10\times \frac{{\sum }_{i=1}^{m}{P}_{i}}{1+{\sum }_{i=1}^{m}{U}_{i}})}^{k-2},$$

where *P*_*i*_ was the number of phased reads at the *i*th phase from the position, *U*_*i*_ was the number of non-phased reads at the *i*th phase from the position, and *m* was the number of phases in the window, and *k* was the number of unique phased siRNAs in the window. *m* was 10 in this study.

### Identification of miRNA/tasiRNA targets in *P. notoginseng*

The putative annotations of target mRNAs were obtained by aligning the sequences of the self-assembled transcript database to the NCBI nr/nt database. The IDs of all mRNA transcripts, as well as the transcripts of primary miRNAs and TAS3, were given in the Supplementary Materials.

The targets of miRNAs and tasiRNAs were predicted with the SeqTar algorithm^23^. For conserved miRNAs, the targets that had less than four mismatches were used for further analysis. The conserved target families with 4 to 5 mismatches were also kept for further analysis if there were no targets in the same family with less mismatches. For novel miRNAs, only targets with at least 1 valid read and less than 4 mismatches were used.

### Validation of miR156g-5p and its targets with qRT-PCR

We collected roots of 16 *P. notoginseng* plants grown in Kunming, Yunnan, China to validate the expression levels of miR156g-5p and two SPL genes targeted by it using the quantitative real-time PCR (qRT-PCR) experiments. The roots collected were frozen in liquid nitrogen immediately. For total RNA isolation, the samples were thawed at room temperature and homogenized in 1 ml of Trizol reagent (Invitrogen, Thermo Fisher Scientific Inc., USA), and an RNA prep pure kit (Invitrogen, Thermo Fisher Scientific Inc., USA) was used according to the manufacturer′s protocol. Then 2 *μ*g of total RNA was reverse-transcribed to cDNA for miRNAs using the miRcute miRNA First-Strand cDNA Synthesis Kit (TIANGEN BIOTECH, Beijing, China). 4 *μ*g RNA was reverse-transcribed to cDNA for target genes using Thermo Scientific RevertAid First Strand cDNA Synthesis Kit (Thermo Scientific, Massachusetts, USA). In the second step, qRT-PCR of the miRNA was carried out using miRcute miRNA qPCR Detection Kit (TIANGEN BIOTECH, Beijing, China) for 120 s at 94 °C, followed by 40 cycles of 20 s at 94 °C and 34 s at 60 °C. And qRT-PCR of the mRNA was carried out using Roche SYBR Green Master mix (Roche, Basel, Swiss) for 120 s at 95 °C, followed by 40 cycles of 15 s at 95 °C and 60 s at 60 °C. The expression levels of *PnACT2* (*P. notoginseng* actin) and U6 were used as the internal controls to standardize the RNA and miRNA samples for each reaction, respectively. Three biological replicates for each experiment were performed. The Ct (2^−ΔΔ*Ct*^) was used to calculate the fold changes^53^. The qRT-PCR primers used in the study were listed in Table S13, and the reverse primers of miR156g-5p and 5.8S ribosomal RNA were provided by miRcute miRNA qPCR Detection Kit. The 16 samples were classified into three groups based on their TRM values using the threshold values determined with the 59 root samples used in the sRNA sequencing profiles. The obtained relative expression levels of miR156g-5p and the two SPL genes were compared for the samples in the Small and Large groups.

### RLM 5′-RACE validations of some identified miRNA targets

Modified 5′-RACE assay was performed using the GeneRacer Kit (Invitrogen) to validate 10 identified targets (as listed in Table S13). Briefly, 5 *μ*g total RNA was ligated with GeneRacer^*TM*^ RNA Oligo (5′-CGACUGGAGCACGAGGACACUGACAUGGACUGAAGGAGUAGAAA-3′) and reverse transcription was performed using SuperScript^*TM*^ III Reverse Transcriptase and oligo dT(18) primer. The resulting cDNA was used as template for PCR amplification with GeneRacer 5′ primer (5′-CGACTGGAGCACGAGGACACTGA-3′) and a gene-specific reverse primer. A second nested PCR was performed using GeneRacer 5′ nested primer (5′-GGACACTGACATGGACTGAAGGAGTA-3′) and a gene specific nested primer. The amplified products were run on a 2% agarose gel, bands with expected size were purified using QIAquick^Ⓡ^ Gel Extraction Kit (QIAGEN) and ligated onto T Vector pMD 19 (Simple)(Takara), following transformation and clone PCR, plasmids were isolated and subjected to Sanger sequencing. Gene specific primers used were listed in Table S13.

### Phylogenetic analysis of TAS3 and tasiRNAs

The phylogenetic trees of the predicted TAS3 loci and their derived tasiRNAs were constructed with the Bootstrap Neighboring-Joining algorithm implemented in ClustalX (version 2.1)^54^ and visualized with TreeView^55^.

### Data availability statement

The 17 *P. notoginseng* small RNA-seq profiles and the *P. notoginseng* degradome sequencing profile were available in the NCBI SRA database under the series accession numbers SRP082250 and SRP087606, respectively. The self-assembled transcript database was available in the DDBJ/EMBL/GenBank database under the accession GFRX00000000. The version described in this paper is the first version, GFRX01000000.

## Electronic supplementary material

Supplementary Information

Table S3

Table S4

Table S5

Table S6

Table S7

Table S11

Table S12

Table S9

Table S10
