# Supplementary material for: Small RNA profiles from Panax notoginseng roots differing in sizes reveal correlation between miR156 abundances and root biomass levels
Source: Sci Rep. 2017 Aug 25;7:9418. doi: 10.1038/s41598-017-09670-8 (PMC5573331; doi:10.1038/s41598-017-09670-8)
Supplement: Supplementary file 1 — Supplementary Information [file 41598_2017_9670_MOESM1_ESM.pdf]

Supplementary Information of Small RNA profiles from *Panax notoginseng* roots differing in sizes reveal correlation between miR156 abundances and root biomass levels

Yun Zheng[1,2,+,*], Kun Chen[1,+], Zhenning Xu[1,+], Peiran Liao[1], Xiaotuo Zhang[2], Li Liu[1], Kangning Wei[4], Diqiu Liu[1,3], Yong-Fang Li[4], Ramanjulu Sunkar[5], and Xiuming Cui[1,3,*]

[1]Faculty of Life Science and Technology, Kunming University of Science and Technology, Kunming, Yunnan, 650500, China
[2]Yunnan Key Laboratory of Primate Biomedical Research; Institute of Primate Translational Medicine, Kunming University of Science and Technology, Kunming, Yunnan, 650500, China
[3]Key laboratory of *Panax notoginseng* resources sustainable development and utilization of state administration of traditional Chinese medicine, Kunming, Yunnan, 650500, China
[4]College of Life Sciences, Henan Normal University, Xinxiang, Henan, 453007, China
[5]Department of Biochemistry and Molecular Biology, Oklahoma State University, Stillwater, Oklahoma, USA
[*]YZ:zhengyun5488@gmail.com and XC:sanqi37@vip.sina.com
[+]These authors contributed equally to this work

Supplementary Tables S1, S2, S8 and S13 are included in this file. Other Supplementary Tables, i.e., Table S3, S4, S5, S6, S7, S9, S10, S11, and S12, are given in separate MS Excel files.

Supplementary Figures S1 to S6 and legends are included in this file.

Table S1. The characteristics of 59 *P. notoginseng* plants used in the study.

No.	Total_Root_Weight (g)	Root_Weight (g)	Height (cm)	Middle_Leave_Length (cm)	Middle_Leave_Width（cm）	Number_of_Complex_Leaves	Leaf_Pattern	Leaf_Number	Total_Leaf_Area (cm^2)	miRNA-seq?	Group
r1	20.7	11.6	57	12.5	4	4	7775	26	30.00		Medium
r2	19.7	14.4	39	12.7	3.7	4	7777	28	28.19	Yes	Medium
r3	6.4	5.4	26	9.2	3.5	2	77	14	19.32		Small
r4	30.9	22.3	42.5	13.6	4.5	4	7777	28	36.72	Yes	Large
r5	11.6	5.8	35	10.4	3	5	77666	32	18.72		Small
r6	24.8	17.2	47	14	4.9	4	7777	28	41.16		Medium
r7	26.8	19.2	55	16.8	4.2	4	7777	28	42.34	Yes	Medium
r8	30.6	23.4	48	14	4.8	4	7777	28	40.32	Yes	Large
r9	43.4	31.1	56	14.8	4.1	5	77777	35	36.41	Yes	Large
r10	11.7	7.8	31	8.5	3.3	5	77777	35	16.83		Small
r12	27.6	19.6	58.5	16	5.5	3	778	22	52.80		Medium
r13	15.2	11.2	38.5	11.3	3.5	4	7777	28	23.73		Small
r14	24.7	13.3	50	13.8	4.2	5	77775	33	34.78		Medium
r15	40.3	29.4	65	15	5.1	4	7778	29	45.90		Large
r16	24.1	16.4	44.5	12	4.2	5	77773	31	30.24	Yes	Medium
r17	30.4	18.7	64	14.2	4.4	5	77775	33	37.49		Large
r18	44.7	30.1	50	16.5	4.6	4	7776	27	45.54		Large
r19	14.7	9.4	28	12.1	3.2	4	7775	26	23.23		Small
r20	11.8	10.1	18	7.2	2.1	5	77775	33	9.07		Small
r21	24.3	17.4	39.5	10.5	3.7	4	7777	28	23.31	Yes	Medium
r22	13.2	8.8	24	6.8	2.4	5	77755	31	9.79		Small
r23	14.6	10.7	27.8	8.3	3	4	7777	28	14.94		Small
r24	34	23.1	54.5	13.8	4.4	4	7756	25	36.43		Large
r25	28.1	19.4	48	15.3	4.6	4	5555	20	42.23	Yes	Medium
r26	16.2	10.3	34	11.5	3.7	4	7776	27	25.53		Small
r27	25.1	20.2	38	12.5	4.2	4	7777	28	31.50		Medium
r28	41	28.8	42.5	14.1	4.9	4	7777	28	41.45	Yes	Large
r29	41.4	28.9	46	14.7	5.9	4	7777	28	52.04		Large
r30	31.4	24.9	55	12.4	3.1	4	7777	28	23.06		Large
r32	15.2	10.5	30	10.3	2.3	4	7776	27	14.21	Yes	Small
r33	17.5	13.5	29.5	11.3	3.8	4	7777	28	25.76		Small
r34	33.6	23.3	49	14.2	5.2	5	77766	33	44.30		Large
r35	16	13.2	24.5	11.4	3.6	4	7777	28	24.62		Small
r36	15.8	10.3	31.5	10.5	2.7	4	7755	24	17.01		Small
r37	26.8	19.3	28	12.3	4	4	7766	26	29.52		Medium
r38	29.3	21.5	54	13.7	4.6	4	7889	32	37.81		Medium
r39	21.7	14	59	12	3.6	5	77745	30	25.92		Medium
r40	24	17.9	31.5	10.9	3.5	5	77777	35	22.89		Medium
r41	33.4	23.1	34	14.5	4.8	4	7775	26	41.76		Large
r42	19.6	14.8	31.5	12.1	3.2	4	7775	26	23.23		Medium
r43	42.2	26.2	45.5	14.2	3.8	4	7777	28	32.38	Yes	Large
r44	24.3	18.1	45	10.3	3.3	4	7776	27	20.39	Yes	Medium
r45	37	29.3	45	13.5	3.5	4	7766	26	28.35		Large
r46	24.5	17	39	13.6	3.8	4	7766	26	31.01	Yes	Medium
r47	19.6	15	36	14.1	4.2	4	7755	24	35.53	Yes	Medium

r48	60.5	44.4	62	17.1	5.5	5	77775	33	56.43	Yes	Large
r49	25.3	18.2	31	11.7	4.3	4	7777	28	30.19		Medium
r50	10.4	7.7	28	9.8	3.6	3	665	17	21.17		Small
r51	32.7	22.3	49	14.3	3.8	5	77777	35	32.60		Large
r52	38.1	23.5	40	14.2	4.2	3	777	21	35.78		Large
r53	16.8	13.3	43	13.2	3.6	4	7745	23	28.51	Yes	Small
r54	9	7.1	32	8.8	3.1	3	555	15	16.37	Yes	Small
r55	16.1	12.1	30.5	10.8	2.8	5	77776	34	18.14		Small
r56	30.1	24.7	48	12.7	4.6	5	77775	33	35.05		Medium
r57	10.2	7.9	30	9.7	3.1	4	7777	28	18.04		Small
r59	14.1	11.2	23	8.6	2.5	6	777665	38	12.90		Small
r60	10.1	7.5	22.5	10	3.3	4	7785	27	19.80		Small
r61	29	17.1	49	13.6	3.6	4	7777	28	29.38		Medium
r62	32.1	22.6	52	11.6	3.4	4	7777	28	23.66		Large

Table S2. The characteristics of *P. notoginseng* plants.

measure	unit	definition
Total_Root_Weight	g	Total root weight was the sum of main root weight, rhizome weight, lateral root weight and fibrous root weight
Root_Weight	g	Root Weight was total root weight minuses the weight of rhizome
Height	cm	The height of the aerial part of a *P. notoginseng* plant, i.e., height of from ground to the flower of the plant
Middle_Leave_Length	cm	The length of largest middle leaves in complex leaves
Middle_Leave_Width	cm	The width of largest middle leaves in complex leaves
Number_of_Complex_Leaves		Number of complex leaves in *P. notoginseng*
Leaf_Pattern		The number of leaves in all complex leaves of a P. notoginseng plant given in a string. For example, 7775 means four complex leaves have 7, 7, 7 and 5 small leaves, respectively.
Leaf_Number		The total number of leaves in all complex leaves of a *P. notoginseng* plant
Total_Leaf_Area	cm^2	The sum of all leaves area in *P. notoginseng*

Table S8. The distribution of degradome reads in different categories of molecules.

category	reads	unique sequences
mRNAs	10,943,534	1,973,725
ncRNAs	1,790,320	18,673
pre-miRBase	3,857	284
Repeats	1,292,747	8,496
total	12,460,345	2,556,355

Table S13. The primer sequences used for the qRT-PCR and the RLM 5'-RACE experiments.

Gene	Sequence (from 5' to 3')	Transcript Accession
qRT-PCR primers		
SPL4-F	TCACATCCTCGGTCACATTC	TR75240|c1_g1_i1
SPL4-R	TACCACCACAACCTTTCTTCA	TR75240|c1_g1_i1
SPL5-F	AACACTTGGCTCTTTGATTGG	TR94274|c1_g1_i1
SPL5-R	CCTCCTAGTTTCCTTGCTTACC	TR94274|c1_g1_i1
PnACT2 -F	TCCAAGGGTGAATATGATGAATCG	TR66434|c0_g1_i1
PnACT2 -R	AACCTCTCCAAAGAGAATTTCTGAGT	TR66434|c0_g1_i1
miR156g-5p-F	GCGCTTGACAGAAGATAGAGAGCAC	TR66267|c0_g1_i1
5.8S ribosomal RNA-F	CGATGAAGAACGTAGCGAAATGC	KT380921 (from NCBI Nucleotide)
RLM 5'-RACE primers		
TR94099Rev1	AGACCTAGCCTATTAAGAGCATATC	TR94099|c1_g2_i1
TR94099Rev2	TGTTGCTCAGTAAGTATTGGCTGAC	TR94099|c1_g2_i1
TR97308Rev1	TGTACACTTTACTGCCATCATCCTC	TR97308|c3_g1_i1
TR97308Rev2	TCAGATGAATGATCTTACCGAATAAC	TR97308|c3_g1_i1
TR101210Rev1	TAGGAAGACAAACTAGACAACATCTC	TR101210|c4_g1_i1
TR101210Rev2	TGTAGAGATTCAGAGTGAAGTAC	TR101210|c4_g1_i1
TR102096Rev1	TCTGTCCCTCCTAGGAAGCTCCTG	TR102096|c6_g1_i4
TR102096Rev2	TCAAGAAGAGCCTGGTTGGATGTG	TR102096|c6_g1_i4
TR98951Rev1	ATAGAGGATGTACCTGAAACCTC	TR98951|c0_g1_i6
TR98951Rev2	TAACGGTGCCTCGGCATCTTCATC	TR98951|c0_g1_i6
TR96826Rev1	AGAAACTGTGCTCATCTGCATCAG	TR96826|c3_g1_i1
TR96826Rev2	TAAGGTGAAGGCTCCATCTGCAAC	TR96826|c3_g1_i1
TR103070Rev1	AAGATCCATGTATTGTCTTCCAC	TR103070|c5_g1_i1
TR103070Rev2	TCAGTTGCAGATCACCGGTATACTG	TR103070|c5_g1_i1
TR53089Rev1	AGCAATGAATCTGAGTAAACTAGTC	TR53089|c0_g1_i1
TR53089Rev2	TGTTCATATAACTCCTGCTAAGGTG	TR53089|c0_g1_i1
TR101352Rev1	ACTACTTACATCCAAGTTGATGTG	TR101352|c7_g2_i2
TR101352Rev2	TAACCACTAGGTAACATGTCAG	TR101352|c7_g2_i2
TR101805Rev1	TATCAGCAATCTCTCTCATGAAG	TR101805|c1_g7_i2
TR101805Rev2	TCGATGACGTGGACGATCATGGAG	TR101805|c1_g7_i2

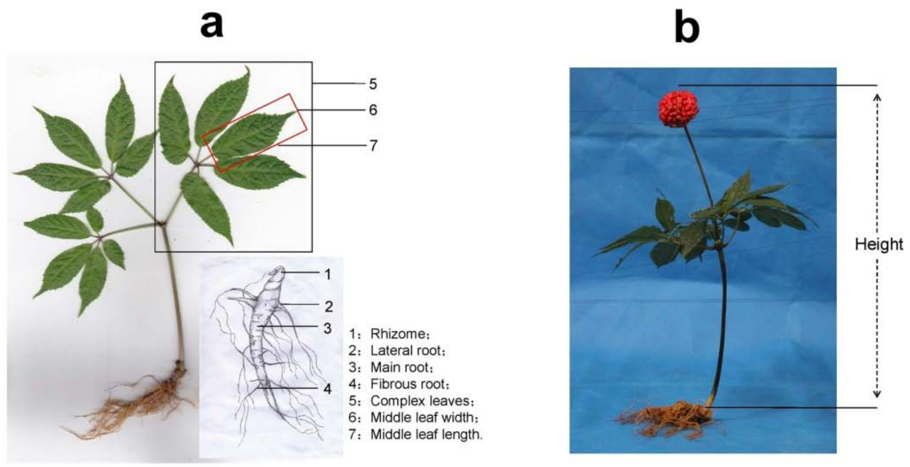

Figure S1. The characteristics of *Panax notoginseng.* (a) The features of leaves and roots of the plant. (b) The height of the plant.

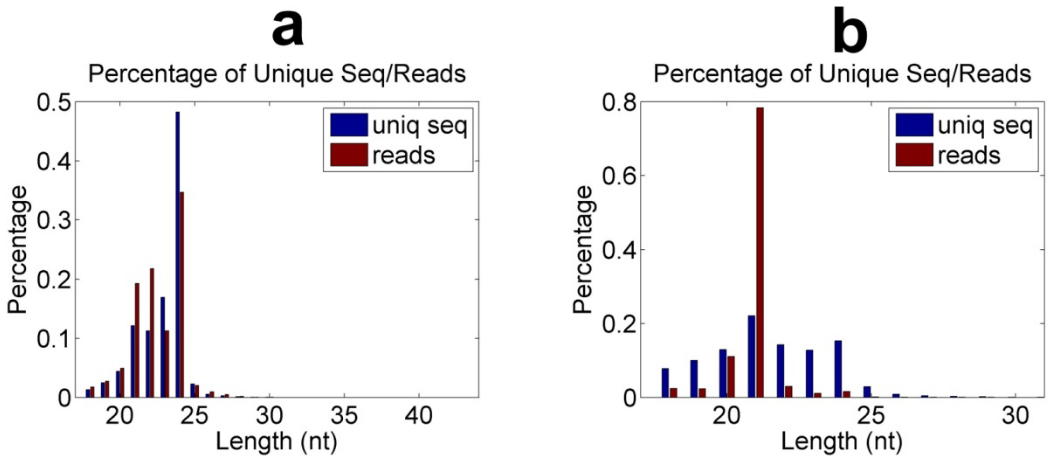

Figure S2. The length distributions of reads and unique sequences in all sequenced reads (a) and reads mapped to pre-miRBase (v21) (b). The vertical axis show the percentages of reads (red bars) and unique sequences (blue bars).

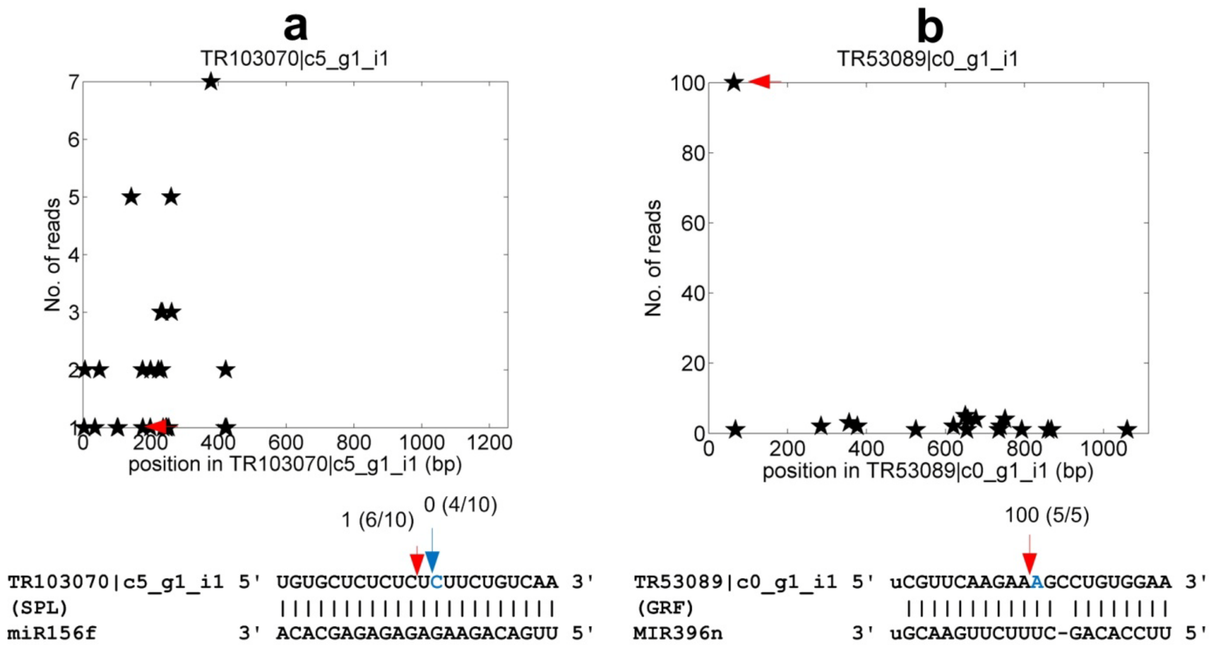

Figure S3. The t-plots and miRNA complementary sites of two targets confirmed by the RLM 5'-RACE experiments. The x-axis is the position on the transcript, and y-axis is the number of degradome reads detected from a position. The arrows in the upper parts correspond to the positions pointed by the arrows of the same colors in the lower parts. The numbers above the arrows indicate the number of degradome reads from the position. The numbers in the parenthesis are the cleavage frequencies determined by the RLM 5’-RACE experiments. (a) miR156f:TR103070|c5_g1_i1 (an SPL gene). (b) MIR396n:TR53089|c0_g1_i1 (a GRF gene).

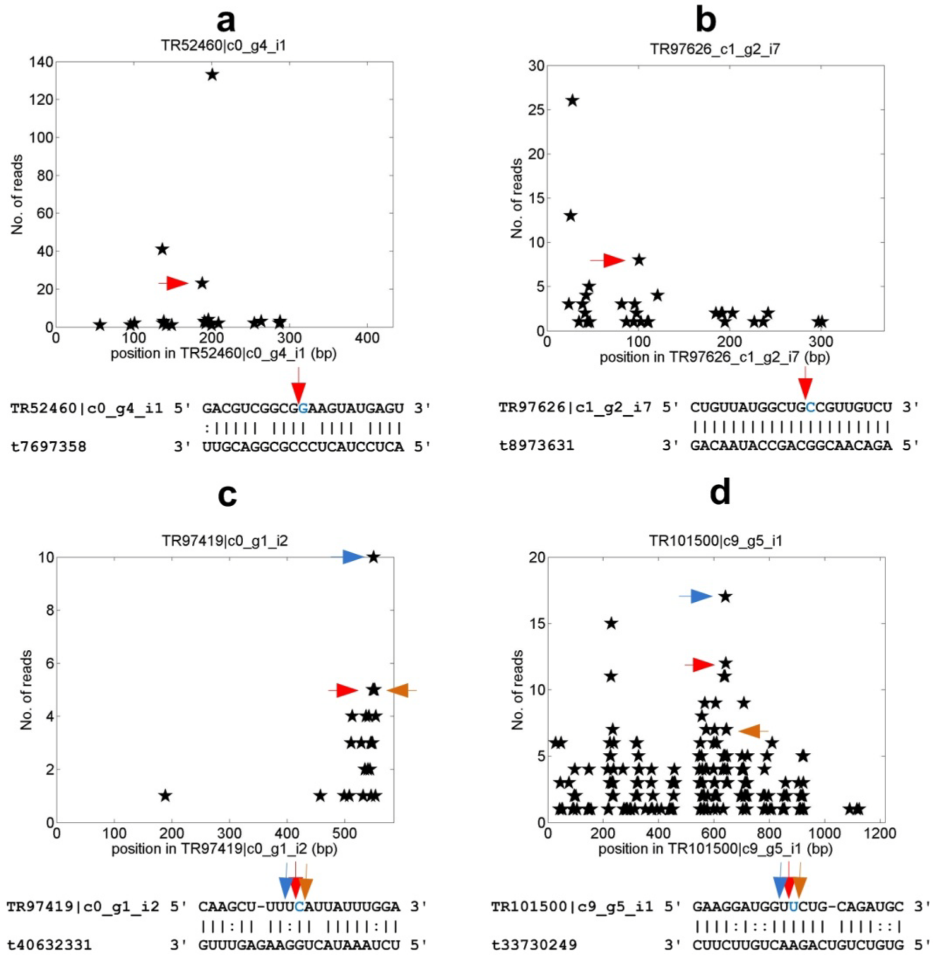

Figure S4. Some predicted targets of novel miRNAs. The x-axis is the position on the transcript, and y-axis is the number of degradome reads detected from a position. The arrows in the upper parts correspond to the positions pointed by the arrows of the same colors in the lower parts. (a) t7697358:TR52460|c0_g4_i1 (a putative 60S ribosomal protein gene). (b) t8973631:TR97626|c1_g2_i7 (an unknown gene). (c) t40632331:TR97419|c0_g1_i2 (an unknown gene). (d) t33730249:TR101500|c9_g5_i1 (a 40S ribosomal protein Sa-2-like gene).

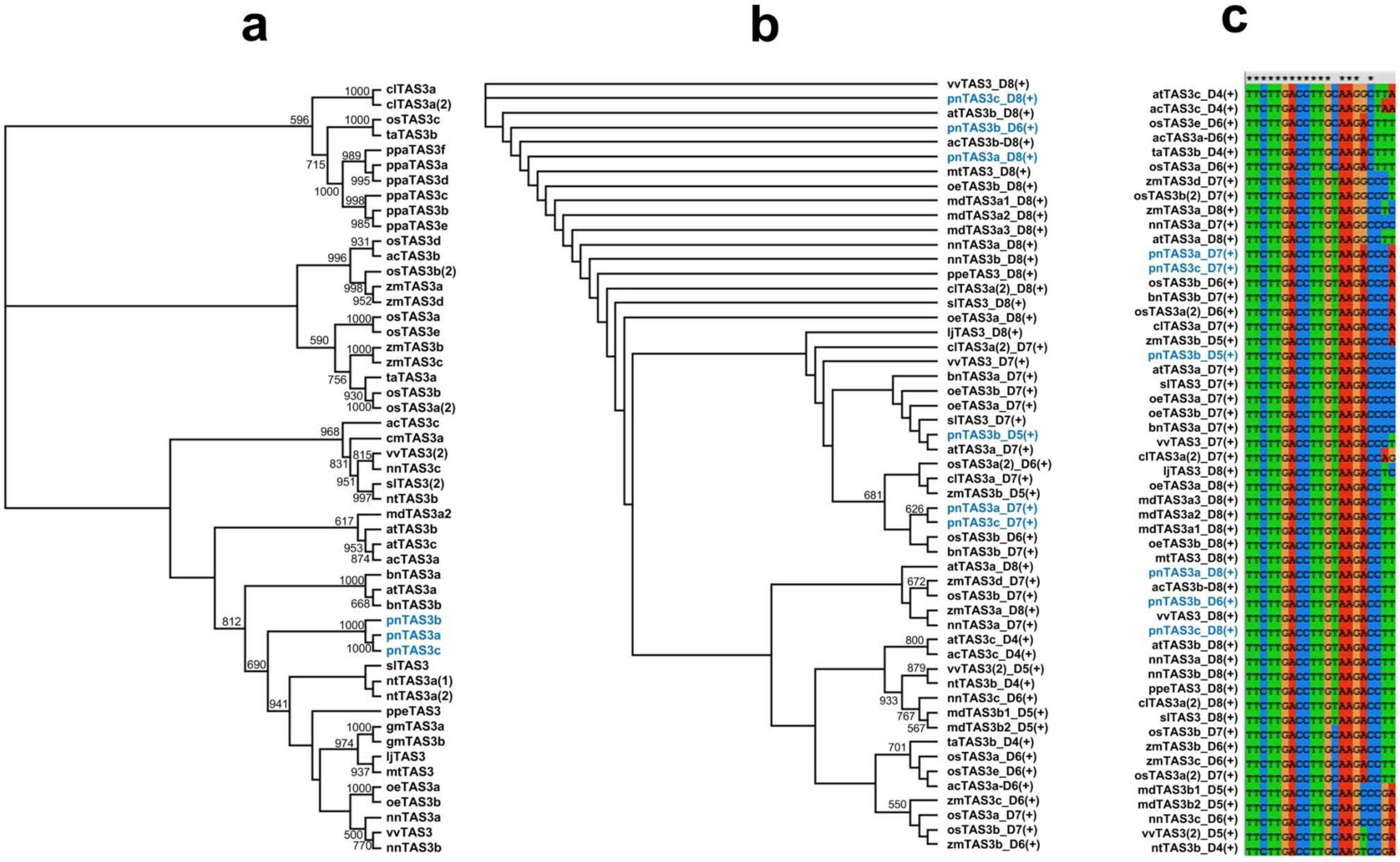
a
clTAS3a
clTAS3a(2)
osTAS3c
taTAS3b
ppaTAS3f
ppaTAS3a
ppaTAS3d
ppaTAS3c
ppaTAS3b
ppaTAS3e
osTAS3d
acTAS3b
osTAS3b(2)
zmTAS3a
zmTAS3d
osTAS3a
osTAS3e
zmTAS3b
zmTAS3c
taTAS3a
osTAS3b
osTAS3a(2)
acTAS3c
cmTAS3a
vvTAS3(2)
nnTAS3c
slTAS3(2)
ntTAS3b
mdTAS3a2
atTAS3b
atTAS3c
acTAS3a
bnTAS3a
atTAS3a
bnTAS3b
pnTAS3b
pnTAS3a
pnTAS3c
slTAS3
ntTAS3a(1)
ntTAS3a(2)
ppeTAS3
gmTAS3a
gmTAS3b
ljTAS3
mtTAS3
oeTAS3a
oeTAS3b
nnTAS3a
vvTAS3
nnTAS3b
b
vvTAS3_D8(+)
pnTAS3c_D8(+)
atTAS3b_D8(+)
pnTAS3b_D6(+)
acTAS3b-D8(+)
pnTAS3a_D8(+)
mtTAS3_D8(+)
oeTAS3b_D8(+)
mdTAS3a1_D8(+)
mdTAS3a2_D8(+)
mdTAS3a3_D8(+)
nnTAS3a_D8(+)
nnTAS3b_D8(+)
ppeTAS3_D8(+)
clTAS3a(2)_D8(+)
slTAS3_D8(+)
oeTAS3a_D8(+)
ljTAS3_D8(+)
clTAS3a(2)_D7(+)
vvTAS3_D7(+)
bnTAS3a_D7(+)
oeTAS3b_D7(+)
oeTAS3a_D7(+)
slTAS3_D7(+)
pnTAS3b_D5(+)
atTAS3a_D7(+)
osTAS3a(2)_D6(+)
clTAS3a_D7(+)
zmTAS3b_D5(+)
pnTAS3a_D7(+)
pnTAS3c_D7(+)
osTAS3b_D6(+)
bnTAS3b_D7(+)
atTAS3a_D8(+)
zmTAS3d_D7(+)
osTAS3b_D7(+)
zmTAS3a_D8(+)
nnTAS3a_D7(+)
atTAS3c_D4(+)
acTAS3c_D4(+)
vvTAS3(2)_D5(+)
ntTAS3b_D4(+)
nnTAS3c_D6(+)
mdTAS3b1_D5(+)
mdTAS3b2_D5(+)
taTAS3b_D4(+)
osTAS3a_D6(+)
osTAS3e_D6(+)
acTAS3a-D6(+)
zmTAS3c_D6(+)
osTAS3a_D7(+)
osTAS3b_D7(+)
zmTAS3b_D6(+)
c
atTAS3c_D4(+)
acTAS3c_D4(+)
osTAS3e_D6(+)
acTAS3a-D6(+)
taTAS3b_D4(+)
osTAS3a_D6(+)
zmTAS3d_D7(+)
osTAS3b(2)_D7(+)
zmTAS3a_D8(+)
nnTAS3a_D7(+)
atTAS3a_D8(+)
pnTAS3a_D7(+)
pnTAS3c_D7(+)
osTAS3b_D6(+)
bnTAS3b_D7(+)
osTAS3a(2)_D6(+)
clTAS3a_D7(+)
zmTAS3b_D5(+)
pnTAS3b_D5(+)
atTAS3a_D7(+)
slTAS3_D7(+)
oeTAS3a_D7(+)
oeTAS3b_D7(+)
bnTAS3a_D7(+)
vvTAS3_D7(+)
clTAS3a(2)_D7(+)
ljTAS3_D8(+)
oeTAS3a_D8(+)
mdTAS3a3_D8(+)
mdTAS3a2_D8(+)
mdTAS3a1_D8(+)
oeTAS3b_D8(+)
mtTAS3_D8(+)
pnTAS3a_D8(+)
acTAS3b-D8(+)
pnTAS3b_D6(+)
vvTAS3_D8(+)
pnTAS3c_D8(+)
atTAS3b_D8(+)
nnTAS3a_D8(+)
nnTAS3b_D8(+)
ppeTAS3_D8(+)
clTAS3a(2)_D8(+)
slTAS3_D8(+)
osTAS3b_D7(+)
zmTAS3b_D6(+)
zmTAS3c_D6(+)
osTAS3a(2)_D7(+)
mdTAS3b1_D5(+)
mdTAS3b2_D5(+)
nnTAS3c_D6(+)
vvTAS3(2)_D5(+)
ntTAS3b_D4(+)

Figure S5. The conservation analysis of TAS3 loci and derived tasiRNAs in *P. notoginseng* and other species. (a) The phylogenetic tree of TAS3. (b) The phylogenetic tree of TAS3 derived tasiRNAs. (c) The multiple sequence alignment of TAS3 derived tasiRNAs generated with ClustalX (version 2.1) [1]. The sequences of TAS3 loci and derived tasiRNAs were used to construct the phylogenetic trees with the Bootstrap Neighbor-Joining algorithm implemented in ClustalX (version 2.1) [1]. Then, the trees were visualized with TreeView [2]. The numbers in the trees are bootstrap values greater than 500 (50%). The lower case letters at the beginnings of the names of TAS3 and tasiRNAs stand for the species, i.e., at (*Arabidopsis thaliana*), bn (*Brassica napus*), cl (*Cunninghamia lanceolata*), cm (*Cucumis melo*), gm (*Glycine max*), lj (*Lotus japonicus*), md (*Malus domestica*), mt (*Medicago truncatula*), nn (*Nelumbo nucifera (Gaertn))*, nt (*Nicotiana tabacum*), oe (*Olea europaea*), os (*Oryza sativa*), ppa (*Physcomitrella patens*), ppe (*Prunus persica*), sl (*Solanum lycopersicum*), ta (*Triticum aestivum*), vv (*Vitis vinifera*), and zm (*Zea mays*). pnTAS3a (TR91608|c0_g1_i1), pnTAS3b (TR91608|c0_g1_i3) and pnTAS3c (Unigene33634_All) are the three TAS3 loci in *P. notoginseng*, respectively. The tasiARFs of TAS3a/b/c in *P. notoginseng* are given in Part c and Figure 6d in the main text.

Supplementary References

1. Larkin,M.A., Blackshields, G., Brown, N.P., Chenna, R., McGettigan, P.A., McWilliam, H., Valentin, F., Wallace, I.M., Wilm, A., Lopez, R., Thompson, J.D., Gibson, T.J., Higgins, D.G. (2007) Clustal W and Clustal X version 2.0. Bioinformatics, 23:2947-2948.
2. Page, R. D. M. 1996. TREEVIEW: An application to display phylogenetic trees on personal computers. Computer Applications in the Biosciences 12: 357-358.

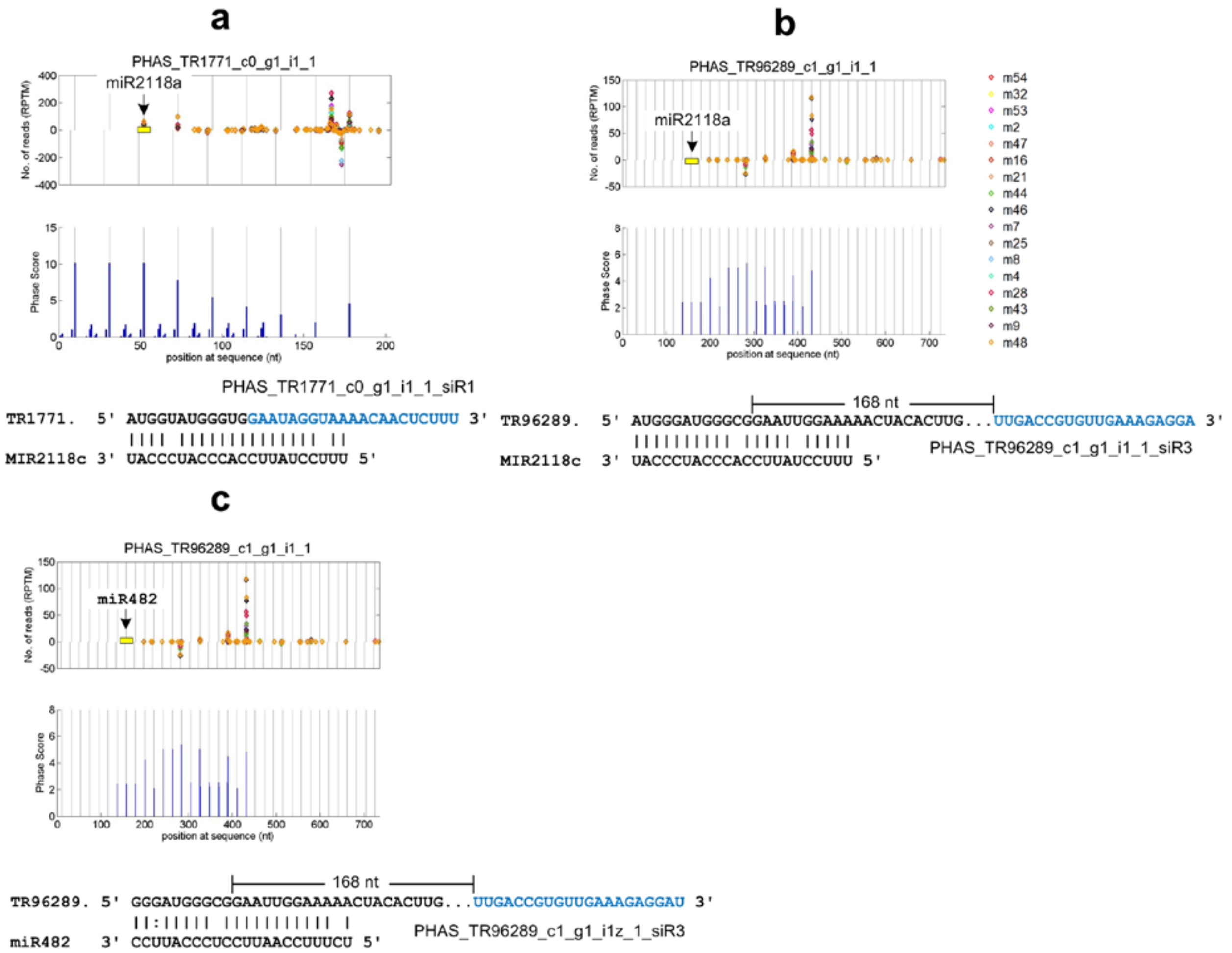

Figure S6. Two putative NB-LRR disease resistance genes (TR1771|c0_g1_i1 and TR96289|c1_g1_i1) that are targeted by MIR2118c and miR482. The diamonds represent the number of 21 nt reads, vertical axis, that appeared at the position of the PHAS loci, horizontal axis, in the 17 small RNA libraries. The vertical gray lines with distances of 21 nt are the phased positions from the position with highest phase scores of the PHAS loci. The yellow boxes in the read distribution panel represent the miRNA complementary sites. Sites pointed by miRNAs from above and under zero read line means miRNAs complement to the plus and minus strand of the predicted PHAS loci, respectively. The predicted miRNA complementary sites are shown below the phase score panel. The blue sequence is one of the 21 nt phasiRNAs that is detected in the small RNA sequencing libraries. (a) TR1771|c0_g1_i1. (b) TR96289|c1_g1_i1 and MIR2118c complementary site. PHAS_TR96289_c1_g1_i1_1_siR3 is 168 nt (eight 21nt phases) downstream of the 10th position of the MIR2118c binding site. (c) TR96289|c1_g1_i1 and miR482 complementary site.
